# Application of Hosomi-Sakurai allylation reaction in total synthesis of biologically active natural products

**DOI:** 10.3389/fchem.2025.1527387

**Published:** 2025-03-28

**Authors:** Justice Akwensi, Robert T. Kumah, Dorcas Osei-Safo, Richard K. Amewu

**Affiliations:** ^1^ Department of Chemistry, School of Physical and Mathematical Sciences, College of Basic and Applied Sciences, University of Ghana, Legon, Ghana; ^2^ Department of Chemical and Petrochemical Engineering, School of Petroleum Studies, University of Mines and Technology, Tarkwa, Ghana

**Keywords:** Hosomi-Sakurai allylation, carbonylation, total synthesis, stereoselectivity, natural products, lewis acid-promotors

## Abstract

The Hosomi–Sakurai allylation reaction has been widely applied in the total synthesis of biologically active natural products, especially in synthesising complex polycyclic compounds containing multi-stereogenic centres since its discovery in 1976. The Hosomi-Sakurai allylation is the allylation of ketones and aldehyde with nucleophilic allylsilanes catalyzed with Lewis acid mainly used to extend the C-C bond in a molecule and also create a new site for manipulation due to the facile transformation of the *pi* (π) bond at the end of its chain. This review highlights only portions of natural product synthetic works that feature the Hosomi-Sakurai allylation reaction or its modification as a key transformation in the synthetic route.

## 1 Introduction

### 1.1 Catalytic allylation reactions

Homoallylic alcohols are a significant class of compounds with extensive applications in the synthesis of biologically active substances. Their unique structural properties make them valuable in various chemical processes, contributing to advancements in medicinal chemistry (Farina et al., 2006; Li et al., 2021; Pellissier, 2016; Shen et al., 2013). Homoallylic alcohols are products of catalytic allylation reactions that employ the use of allylic reagents under mild reaction conditions. Allylation reactions are particularly advantageous due to their use of cost-effective, nontoxic, and stable nucleophiles such as allyltrialkylsilanes, allyltrialkoxysilanes among others. Although significant advancements have been made in allylation reactions, only a limited number of catalytic asymmetric allylation reactions have been reported. This is particularly interesting due to the importance of chiral tertiary homoallylic alcohols in organic synthesis. (Fleming et al., 1989; 1997; Langkopf and Schinzer, 1995; Lee, 2020).

Allylation of carbonyl compounds is one of the most used methods of forming C-C bonds in the most versatile manner (Lee, 2020). In 1964, Mikhailov and co-workers reported the first reaction between an allyl-metal and a carbonyl catalysed by triallylborane (Stepanyan et al., 2001). A number of allyl-(semi)-metals such as Si, Mn, Sn, Sb, Mg, Bi, Al, B, Cr, Li, Zn, Ti, Zr and In have been applied in carbonyl allylation in the total synthesis of simple and complex compounds (Bols and Skrydstrup, 1995; Denmark and Fu, 2003; Dilman and Ioffe, 2003; Fleming et al., 1989; 1997; Langkopf and Schinzer, 1995; Lee, 2020; Masse and Panek, 1995; Stankevich and Cook, 2023; Yamamoto and Asao, 1993). Allylation of carbonyl compounds to produce corresponding alcohols and enols is a well-established synthetic method that employs allylic reagents in the presence of Lewis acids catalysts to form C-C bonds and alcohol derivatives in high-yields (Yamamoto and Asao, 1993). Several other allylation reactions including the Tsuji-Trost allylic reaction, Keck radical allylation, Hosomi-Sakurai allylation reactions among others have been reported in the past 2 decades. For example, the Tsuji-Trost allylic reaction is a versatile and high-yielding reaction in this category. The reaction involves the use of Pd (0) to catalyze the allylation of several nucleophilic species, including methylenes, enolates, amines, and phenols (Li, 1999; Song et al., 2007). These nucleophiles are combined with allylic compounds, such as allyl acetates and allyl bromides, (Supplementary Scheme S1a) (Hegedus et al., 1978; Keck and Yates, 1982; Trost and Van Vranken, 1996; Tsuji, 1969). Tsuji-Trost allylic reaction is applicable in the total synthesis of indole alkaloids such as Desethylibogamine, Isoquinuclidine and (±)-Catharanthine (Trost, 2015; Trost et al., 1978; Tsuji, 1986a; 1986b; Tsuji and Takahashi, 1965). Keck radical allylation is also frequently used in C-C bond formation (Supplementary Scheme S1b) (Aiqin et al., 2011; Keck and Yates, 1982). Keck radical allylation proceeds through the generation of radicals from alkyl bromides, chlorides, phenyl-selenides, and thioacylimidazoles in the presence of a radical initiator such as azobisisobutyronitrile (AIBN) and benzoyl peroxide (BPO) (Sumino et al., 2018). The application of Keck allylation is limited by the challenges associated with the purification of the corresponding products and the inherent toxic nature of organotin reagents used in the reaction (Supplementary Scheme S1b) (Fent, 1996).

**SCHEME 1:**
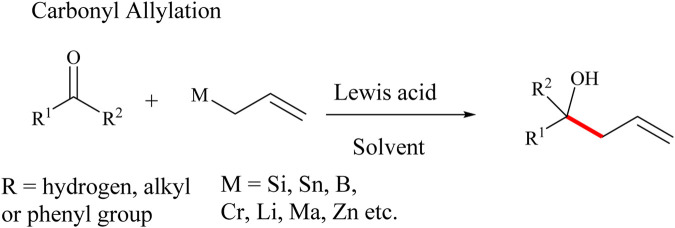
A typical example of an HSR (allylation of carbonyl compounds) reaction catalyzed by Lewis acid to give the corresponding alcohols.

The Hosomi-Sakurai allylation reaction (HSR) is the type of allylation reaction that efficiently creates C-C bonds in the total synthesis of optically active natural products (Scheme 1) (Majetich et al., 1990). HSR involves the allylation of carbonyl compounds using allylic reagents in the presence of Lewis acid catalysts to give the corresponding alcohols in good yields. The HSR usually gives quantitative yields and hence produces fewer by-products. The substituents of the allylsilane are useful in controlling the stereochemistry of the products although the reaction is not centered on the silicon atom in most cases except for chiral allysilanes which usually yield the chiral product (Chan and Wang, 1992; Kira et al., 1990).

HSR is also applicable in the synthesis of polymers by repetitively allylating dialdehydes with bisallylsilanes to give a hydroxyl and olefinic polymer with repeating units (Supplementary Scheme S4) (Itsuno, 2005). A typical example is the preparation of Si-phenyl linkage polymers using HSR in the presence of TBAF/THF overnight (Supplementary Scheme S4). However, low molecular weight monomer units of homoallylic alcohols with high enantiomeric purity were reported (Kumagai and Itsuno, 2000).

**SCHEME 2:**
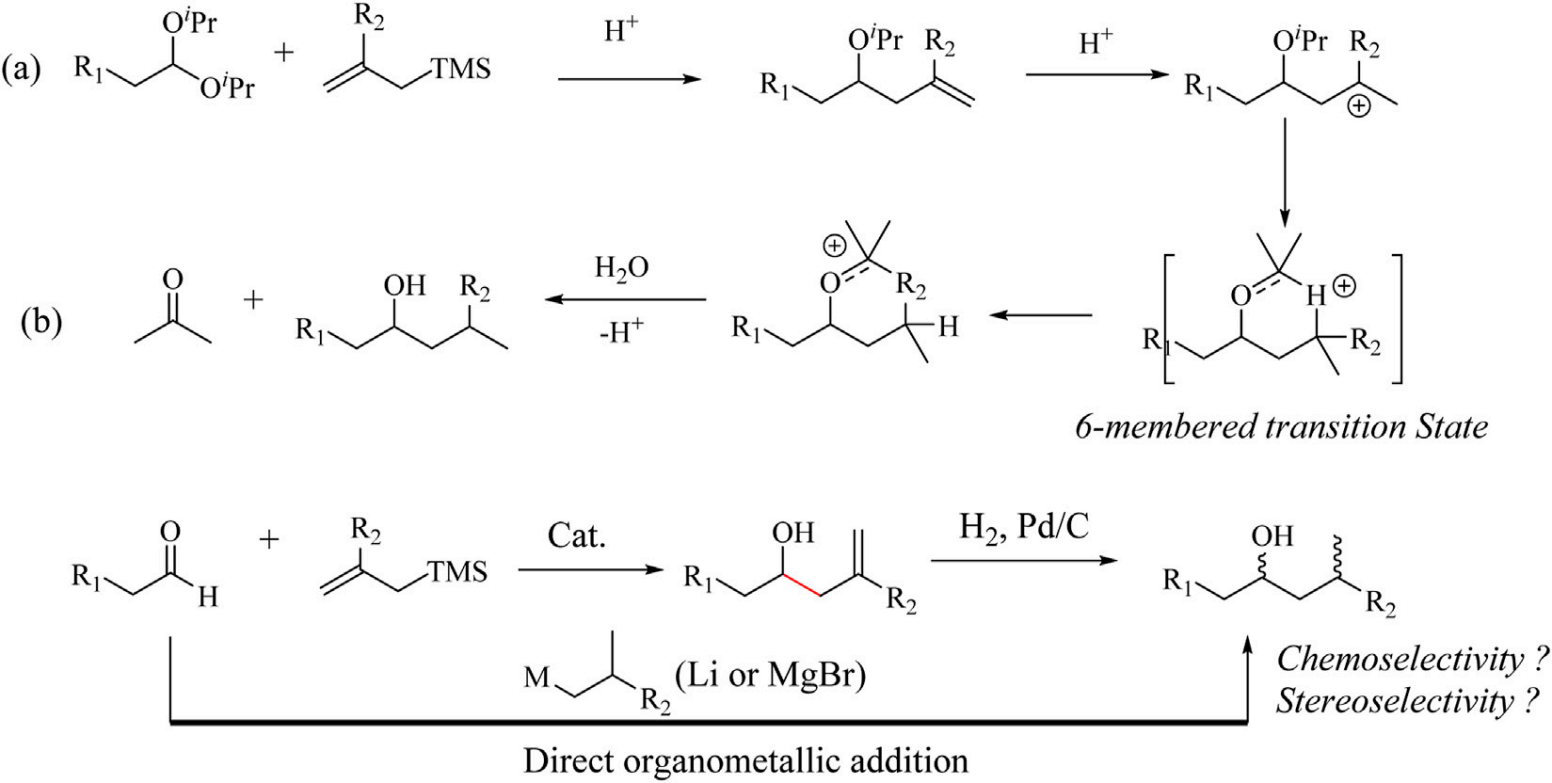
Reductive Hosomi-Sakurai allylation reaction **(a)** Intramolecular 1,5-hydride transfer rearrangement and **(b)** direct Grignard reaction.

Bauer and co-workers discovered a reductive HSR of acetals (Bauer and Maulide, 2018) through the 1,5-hydride transfer transformations (Scheme 2a). The reaction exploits *in situ* generation of homoallylic ether after the HSR with the acetal substrate. Subsequent protonation to generate a carbocation at the γ-position to the ether group leads to an intramolecular 1,5-hydride transfer rearrangement to form an oxocarbenium ion. The addition of water leads to the formation of the corresponding alcohol product. The reaction mechanism yields a similar product to a direct Grignard/Alkyl-Li reagent addition to an aldehyde (Scheme 2b). Air- and water-sensitive Grignard reagents bearing functional groups such as α,β-unsaturated esters, and alkyl bromides are tolerated under these reaction conditions (Bauer and Maulide, 2018). However, the reaction is not applicable when carbonyl substrates bearing aromatic groups, unprotected alcohols, and thioethers are used, as illustrated in Supplementary Scheme S2. In addition, the homo-ether intermediate formed in the HSR could subsequently yield the desired homoallylic products with phenyl-allylsilane if the amount of catalyst was reduced to 2.7 mol%, (dr = 4.5:1) (Bauer and Maulide, 2018). It is interesting to note that this reaction was not only chemoselective but also stereoselective when pro-chiral carbonyl substrates were used (Scheme 2).

### 1.2 History of Hosomi-Sakurai allylation reactions

The HSR, commonly referred to as the Sakurai allylation or Sakurai reaction, was discovered in the 1970s as an alternative to the classical allylation method of C-C bond formation and has been extensively studied over the years (Bauer and Maulide, 2018). The HSR of carbonyl compounds and their derivatives with the allyl trialkylsilane as the allylating agent in the presence of a Lewis acid as a catalyst, proceeds rapidly even at an extremely low temperature of −78°C (Hosomi and Sakurai, 1976). The H-SR is applicable in the allylation of aliphatic, alicyclic, and aromatic carbonyl compounds using allylmethylsilane and TiCl_4_ as a catalyst. Allylsilanes are reported to be weakly nucleophilic hence the Lewis acid catalyst is used to activate and increase the reactivity of the carbon electrophile in the solution. This results in a C-C bond formation that occurs only at the γ-position to the silicon moiety (Yamamoto and Sasaki, 1989).

Allylsilanes are very useful in organic synthesis because they are easy to handle, can be used at room temperatures, and do not require special storage conditions. Their reactions are usually smooth (homogeneous) with different electrophiles under Lewis acid conditions. On the contrary, other allylating agents such as allyl-magnesium halides, -Li, -Cu, and -Ti compounds require specific temperatures and moisture-free working atmosphere and reaction conditions (Yamamoto and Sasaki, 1989). The major advantage of the HSR is that allylsilanes are readily available inexpensive starting materials. They are stable, have low toxicity, compatible with multiple functional groups, and chemically inert to atmospheric conditions (Fleming, 1991; Hosomi, 1988; Yamamoto and Asao, 1993). However, the reaction depends on the presence of Lewis acids such as TiCl_4_, BF_3_⋅OEt_2_, SnCl_4_, and EtAlCl_2_ as activators because allylsilanes do not readily react with non-activated electrophiles (Lade et al., 2017).

### 1.3 Mechanism, scope and features

**SCHEME 3:**
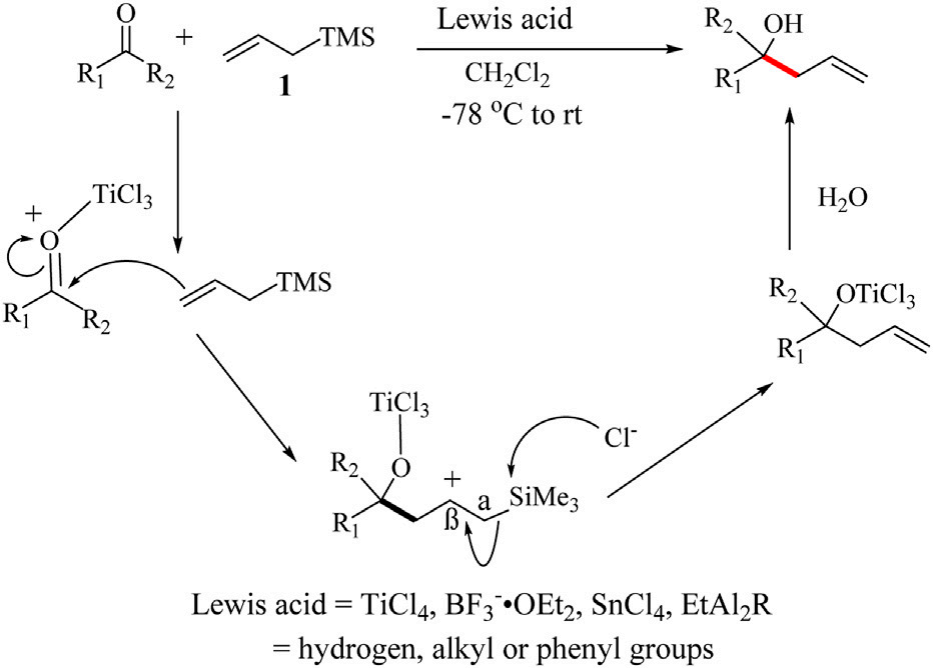
General reaction and mechanism of the HSR (Kira et al., 1990).

A typical reaction pathway of the HSR is illustrated in Scheme 3. An oxocarbenium ion is first generated from the reaction of the carbonyl compound with the Lewis acid which makes the carbonyl carbon more electrophilic. It begins with a nucleophilic attack of the γ-carbon of the allylsilane, **1** on the carbonyl to generate a carbocation which is stabilized by the silyl group (Schweig et al., 1974). The α-Si-C bond at this stage is weak and can be easily cleaved to neutralize the carbocation, hence forming the alkene. Halide from the catalyst facilitates the facile cleavage to form the corresponding intermediate (Ponomarenko & Mironov, 1954). The reaction is usually completed by the addition of H_2_O to give the desired allyl-alcohol products (Scheme 3). The propylene unit created through a reaction between an aldehyde and allylic reagents is very useful in the synthesis of optically active molecules. One of the key features of silyl groups is the ability to stabilize charges on their immediate. Specifically, this includes stabilizing geminal anions (α-effect) and vicinal cations (β-effect). The α-effect is often observed through the metalation of silanes in the α-position using strong bases (Britten et al., 2021). However, the stabilizing influence of the silyl group is significantly more pronounced for the β-silicon effect compared to the α-effect (Roberts and McLaughlin, 2022).

Under Lewis acid conditions, it was observed that the reaction proceeds through a less open cyclic transition state, and more asymmetric products could be achieved (Kira et al., 1990). The HSR is regioselective due to the formation of a stable silylcarbocation intermediate (Hosomi et al., 1978a). In 1978, tetra-*n*-butylammonium fluoride (TBAF) was introduced as an alternative catalyst to Lewis acids. TBAF reacts with allylsilanes to give a stable Si-F intermediate which attacks carbonyl leading to the formation of silyl ethers (Hosomi et al., 1978b). TBAF is also effective in catalysing allylation of synthetic intermediates such as acetals, aldimines/imines/iminium ions (Shingo et al., 2002), aldehydes, acids chlorides (Yadav et al., 2003), epoxides (Yamasaki et al., 2002), ketals, keto-imines, ketones, oxocarbenium ions (Hosomi et al., 1978a) and α,β-unsaturated carbonyls (Lade et al., 2017; Roberts and McLaughlin, 2022). Allylation of α,β-unsaturated carbonyl compounds, however, generates the ketone-enol tautomers instead of the alcohol which continues the allylation cycle as illustrated in Supplementary Scheme S2 (Fürstner and Voigtländer, 2000). A number of Lewis acids have been reported as pre-catalysts in HSR in the past 3–5 decades. Lewis acid reagents such as SnCl_4_, AlCl_3_, BF_3⋅_OEt_2_, Me_3_SiOTf, Me_3_SiI, and Me_3_O^+^BF_4_^−^ or their combinations have also been used (Birch et al., 1981; Colvin, 1978; Hosomi et al., 1984; Nishiyama et al., 1982; Noyori et al., 1981; Yotsu-Yamashita et al., 1995).

**SCHEME 4:**
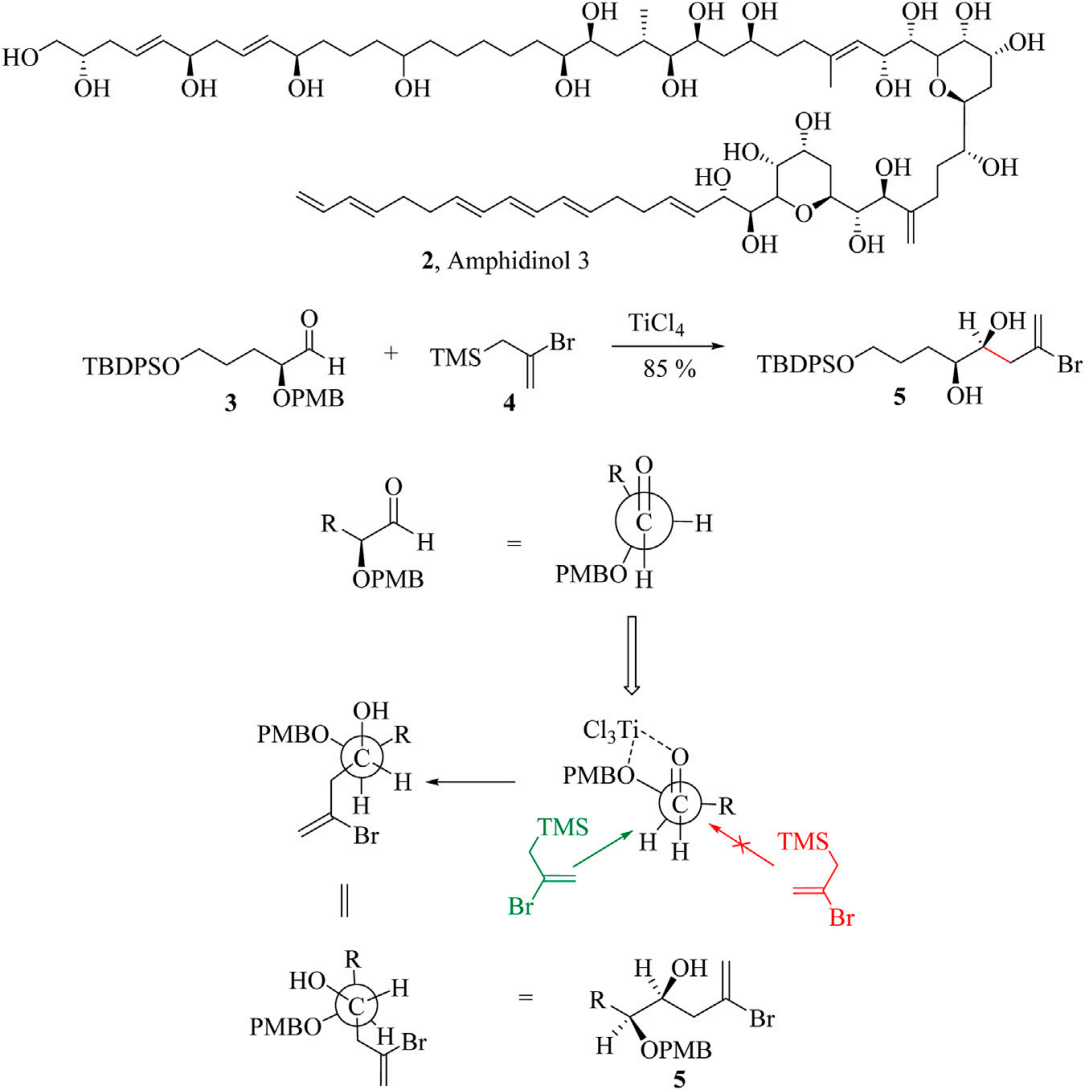
An illustration of the stereospecificity of HSR.

Despite the numerous applications, the use of Lewis acid catalysts in HSR is limited by the inability to recover and reuse them in the subsequent reaction cycles (Supplementary Scheme S3). In addition, Lewis acids are susceptible to degenerate to allylic metal oxides and are therefore added in stoichiometric amounts to ensure the completion of the reaction (Mahlau and List, 2013). The HSR typically yields *syn*-addition products, making it highly diastereoselective in the allylation of carbonyl compounds. The Newman projection (Scheme 4) of the allylation of aldehyde **3** with (2-bromoallyl) trimethylsilane **4** to form **5**, a key intermediate in the synthesis of amphidinol 3, (**2**) demonstrates the diastereospecificity of the HSR. In the Newman projection, the intermediate coordinated to the titanium tetrachloride with the two oxygen atoms tethered together, fixing the conformation of the substrate. This is known as the Cram chelation control mechanism (Cram and Elhafez, 1952; Stanton et al., 2010). The (2-bromoallyl) trimethylsilane nucleophile then approaches along the Bürgi-Dunitz angle, but only one side of the approach is possible as the other is hindered by the R groups. This ensures the formation of only one diastereomer in high yields. Amphidinol 3 (**2**) is a potent antifungal compound produced by the dinoflagellate Amphidinium klebsii (Grisin and Evans, 2015; Huckins et al., 2007; Oishi, 2024; Wakamiya et al., 2020).

### 1.4 Drawbacks of the HSR

The HSR typically proceeds through an open-chain transition state mechanism, which inherently limits the scope of the method. Since carbonyl compounds are not highly reactive, there is a need to increase their reactivity by using strong Lewis acids as catalysts or by activating the allylsilane, especially those with electron-withdrawing groups (Mahlau and List, 2013). However, this approach is not favourable for late-stage functionalization, as it can be intolerant of functional groups and lead to loss of regioselectivity in some cases, resulting in low yields (Chemler and Roush, 2000). Efforts to increase the electrophilicity of carbonyl compounds often lead to a loss of regioselectivity when the allylsilane is activated by a fluoride source (Denmark and Fu, 2003). While the open anti-periplanar transition state leads to the formation of the *syn*-product in most cases, it also impedes the enantioselectivity of the mechanism (Denmark and Almstead, 1994).

### 1.5 Modifications to the HSR

Attempts to address the challenges of HSR have led to the discovery and use of a chiral base catalyst (**S44**), transition metal fluoride (**S45**), chiral Lewis acids (**S47**) Supplementary Figure S1, and tartrate-modified allylsilanes to improve the selectivity and versatility of the HSR (Denmark and Fu, 2001; Gauthier and Carreira, 1996; Ishihara et al., 1993; Malkov et al., 2002; 2008; Schäfers et al., 2022; Zhang et al., 1997). Due to the inherent challenges associated with the HSR, Schäfers et al. developed a dual catalytic system that uses less basic allyl chromium instead of allylsilanes (Schäfers et al., 2022). This reaction proceeds through a ring-closed Zimmerman−Traxler transition state, initiated using the sterically hindered acridinium photocatalyst and blue light-emitting diode **(**LED) irradiation (Supplementary Scheme S13). These reaction conditions often yield undesirable homoallylic products with different chemo- and diastereoselectivity. Mechanistic analysis showed that the reaction proceeds through a cyclic mechanism, leading to the formation of the *anti*-product.

This alternative approach using allyl chromium and photocatalytic activation represents a valuable strategy to overcome the inherent limitations of the HSR, providing access to complementary stereochemical outcomes (Schäfers et al., 2022). The HSR has seen numerous modifications to its reaction conditions since its discovery. For example, new chiral catalysts were introduced with allyltrialkylsilanes, allyltrialkoxysilanes, or allyl trichlorosilane to attain enantiomerically pure allylated products (Kaib et al., 2016; Kampen et al., 2008; Lee, 2020; Sai and Yamamoto, 2015; Schreyer et al., 2019).

The allylation of ketones to generate homoallylic alcohols through the HSR has been a challenge and has proven unsuccessful over the years. Despite these challenges, several asymmetric allylations of ketone have been reported (Niwa et al., 2019; Pellissier, 2006; Tietze et al., 1995; 2004; Wadamoto et al., 2003). The most efficient one is the use of fluoroallylsilane derivatives and Pd-based catalysts instead of the usual Lewis acids (Hayashi et al., 2010). Bismuth bromide has also been reported as a useful catalyst in the allylation of carbonyls by Komatsu and co-workers. Allylation with bismuth bromide is compatible with trimethylsilyl, trifluoromethane sulfonate (TMSOTf) (Noyori et al., 1981), and trimethylsilyl iodide (TMSI) (Komatsu et al., 1997).

Alternatively, Sakurai-Hosomi-Yamamoto allylation has been an effective approach for the stereoselective synthesis of a number of chiral homoallylic alcohols in high yields by employing Chiral (*R*)- and (*S*)-BINAP.AgF catalysts (Supplementary Scheme S8) (Makita et al., 2003; Mirabdolbaghi and Dudding, 2012). Although the reaction mechanism for the asymmetric (*R*)- or (*S*)-BINAP.AgF Sakurai-Hosomi-Yamamoto allylation reaction remains unknown, Mirabdolbaghi et al., proposed a transition state (TS1) that rationalized the enantioselectivity of the mechanism as shown in Supplementary Scheme S8 (Mirabdolbaghi and Dudding, 2012).

Yamamoto and his coworkers further disclosed the synthesis of the *γ-* and *anti*-products upon using AgF and a chiral bisphosphine catalyst such as (*R*)-DIFLUOROPHOS (Nokami et al., 2003; Wadamoto et al., 2003). This reaction is also assumed to progress through the cyclic transition state that is generated *in situ* from the transmetalation of allylsilanes to AgF (Supplementary Scheme S5). This modified Ag-catalyzed asymmetric allylation reaction is amenable to ketone as well as aldehydes (Komiyama et al., 2017; Wadamoto et al., 2003).

**SCHEME 5:**
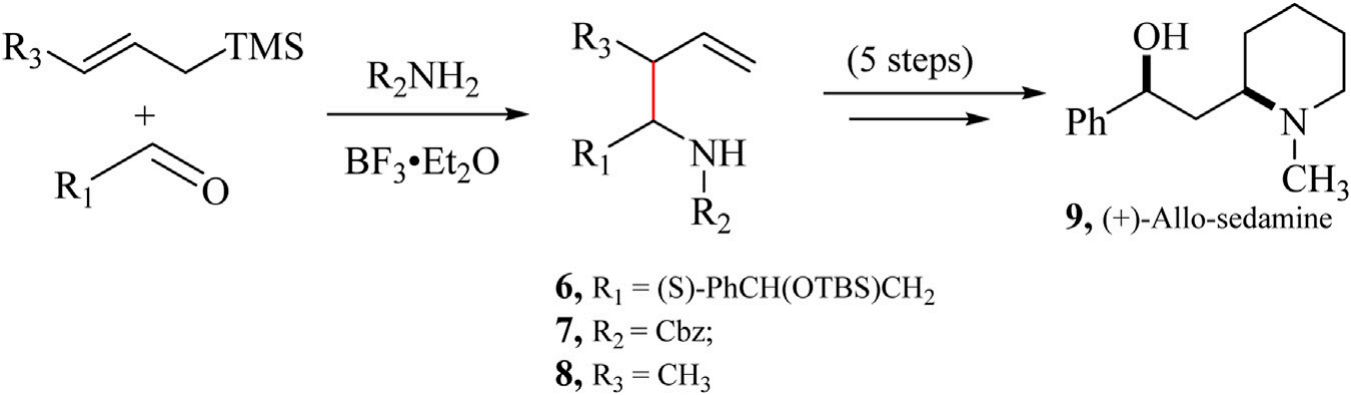
Modified Aza-Hosomi-Sakurai (aHS) allylation reaction for synthesizing (+)-Allo-sedamine (**9**) (Girard, 2015).

The aza-Hosomi-Sakurai (aHS) reaction, developed by Veenstra and Schmid (1997), is an additional variation of the HSR. This method allows for the one-step synthesis of homoallylamines which involves reacting an allylsilane with an aldehyde, a carbamate, or a sulfonamide, in the presence of a Lewis acid catalyst (Scheme 5). This reaction mechanism permits the synthesis of *N*-protected homoallylamines, which can be utilised to make *N*-heteroatomic rings in a few steps (Ella-Menye et al., 2005) leading to the synthesis of pyrrolidine and piperidine rings and eventually (+)-Allo-sedamine (**9**) (Girard, 2015).

Onishi et al. (2002), developed a highly effective Lewis acid by combining InCl_3_ with Me_3_SiCl. The hybrid catalytic system is effective for HSR and Friedel-Crafts allylation and hydrosilylation reactions. Hexafluoroisopropanol (HFIP) has also proven to be an efficient source of a hydrogen bond donor and as a catalyst in the form HFIP–DCM (4:1 (v/v)) solution (Motiwala et al., 2022). To form the desired targeted allylated aldehyde and acetal products product, HFIP (2–5 eq.) was required (Supplementary Scheme S6) (Berkessel et al., 2006; Motiwala et al., 2022). HSR of hydroxy-aldehydes with allytrifluorosilanes was discovered to be stereospecific in the presence of triethylamine and chiral Lewis acid catalysts (Kira et al., 1990). Asymmetric Hosomi-Sakurai reactions (AHSR) could be effectively achieved with *ee* up to 92% using chiral Lewis (Denmark et al., 1994; Iseki et al., 1999; Wang et al., 1999).

Furthermore, a new trityl tetrakis (pentafluophenyl) borate [(Ph_3_C)[BPh(^F^)_4_] has been discovered as a catalyst to mediate HSR of β,γ-unsaturated α-ketoesters to yield γ,γ-disubstituted α-ketoesters in excellent yields using allylsilane reagent (Chien et al., 1991; Hollis and Bosnich, 1995; Kosugi et al., 1985; Mukaiyama et al., 1984). HSR of crotyl geminal bis(silane) with aldehydes (Supplementary Scheme S7) for instance can be carried out more efficiently by using the (Ph_3_C)[BPh(^F^)_4_] catalyst (Chu et al., 2017; Santos and Silva, 2018).

## 2 Applications of HSR in total synthesis of biologically active natural products

A plethora of synthetic methods have been studied and advanced in the synthesis of complex compounds including natural products over the past decades. Among them is HSR of carbonyl intermediates which provides a straightforward and versatile method for preparing these compounds. The HSR is characterised by the formation of a new carbon-carbon bond with high functional group compatibility. The stereoselectivity of the reaction is largely regulated by substituents on substrates under mild reaction conditions. The use of allylsilanes makes this allylation a useful tool for synthesizing complex natural products. Recently, Lee (2020) reported the application of Hosomi-Sakurai in the total synthesis of a number of natural products. In this review, we will focus only on selected examples of total synthesis of biologically active natural products that have utilized HSR as a key transformation method in the past 2 decades and were not included in Lee work.

### 2.1 Total synthesis of aburatubolactam A

Aburatubolactam A (**10**) is a natural product isolated from the culture broth of marine mollusc bacterium *Streptomyces* sp., SCRC-A20 in Japan (Bai et al., 2016; Henderson et al., 2008). Uemura and co-workers determined the complete structure and absolute stereochemistry of the natural product using X-ray crystallographic analysis (Bae et al., 1996). Biological screening of Aburatubolactam A (**10**) exhibited cytotoxicity properties against human cancer cells by inhibiting cell proliferation, anti-microbial activity, and the inhibition of superoxide generation (Israr et al., 2024). Aburatubolactam A (**10**) has been observed to be an inhibitor of protein kinase C in both rat brain tissue samples and rat liver cell membrane preparations (Israr et al., 2024). Additionally, Aburatubolactam A (**10**) has been found to suppress the growth of *Mycobacterium tuberculosis* bacteria by disrupting the synthesis of DNA, RNA, and proteins within the bacterial cells (Henderson et al., 2008).

**SCHEME 6:**
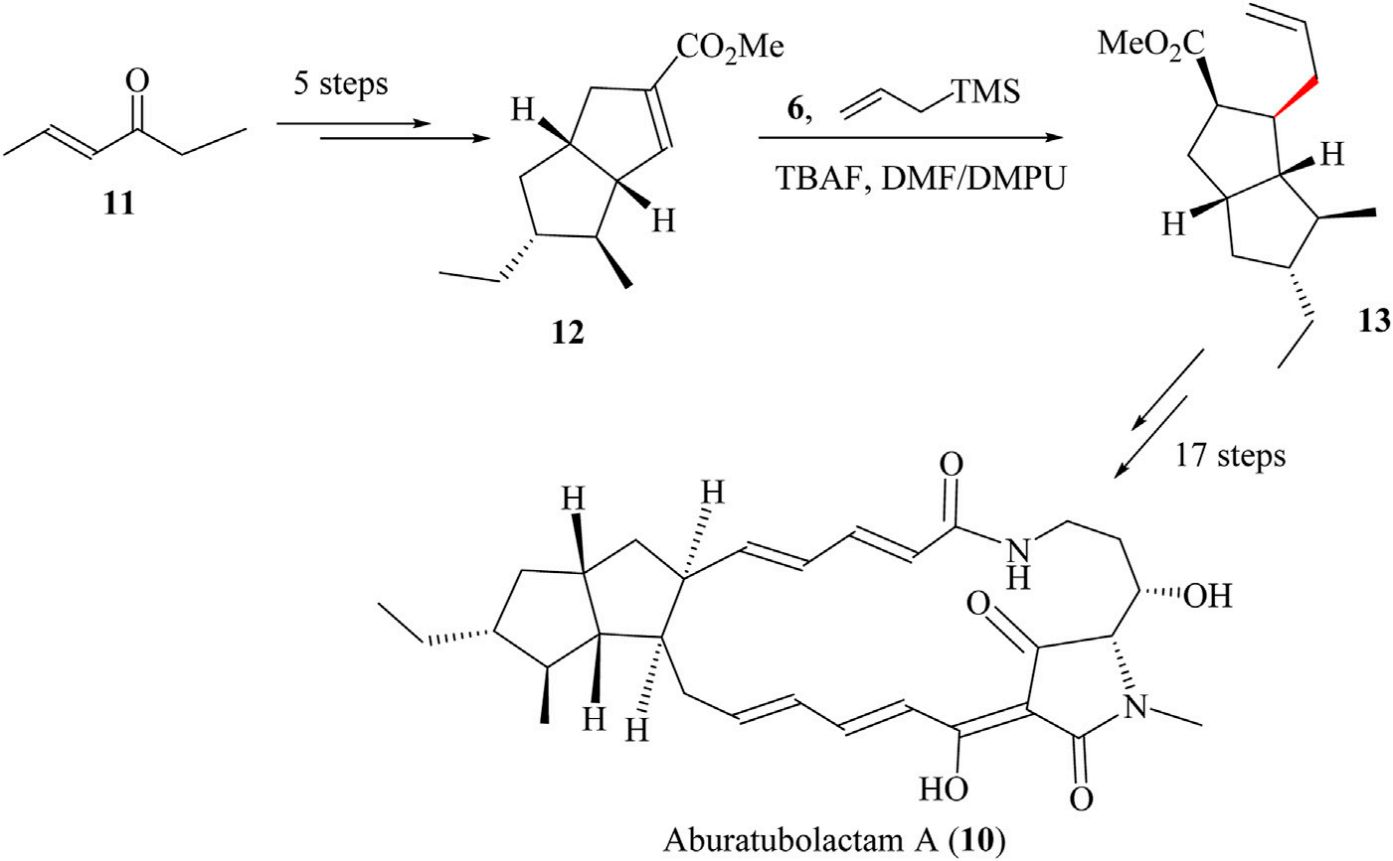
Total synthesis of Aburatubolactam A (**10**) (Bai et al., 2016).

A total of 23 steps were required to synthesise Aburatubolactam A (**10**) starting from commercially available ketone **11**, followed by five steps to form bicyclo [3.3.0] octene **12** with a 90% yield (Scheme 6) (Henderson et al., 2008). The fluoride-mediated Sakurai allylation using TBAF in a solvent mixture of dimethylformamide and *N,N′*-Dimethylpropyleneurea (DMF-DMPU) resulted in the formation of intermediate **13** (Majetich et al., 1986). This intermediate was obtained as a 4:1 mixture of inseparable diastereomers, achieving a yield of 78%. This ratio was then improved in favour of the desired isomer to 2:1 by the protonation of the silylketene acetyl derived from **13**. The resulting product was taken through 11 other sequential steps to achieve Aburatubolactam A (**14**) in 46% total yield (Bai et al., 2016; Henderson et al., 2008) (Scheme 6).

### 2.2 Total syntheses of (−)-acutumine

(−)-Acutumine **14**, is an alkaloid isolated first from the roots of *Sinomenium acutum* in 1929 by Goto and co-workers (Li et al., 2009). It has also been discovered in *Hypserpa nitida* and *Apis cerana*. The family of (−)-Acutumine has structural similarities to the Hasubanan family of alkaloids such as (−)-Dechloroatunine (**15**), and (−)-Hasubanonine (**16**). (−)-Acutumine (**14**) inhibits the proliferation of human T-cells and memory-enhancing properties in the Wistar rat model (Yu et al., 2002). The structure complexity with embedded chirality in the spirocyclopentenone rings 1 and 2 of (−)-Acutumines poses difficulty in its total synthesis (King et al., 2013).

**SCHEME 7:**
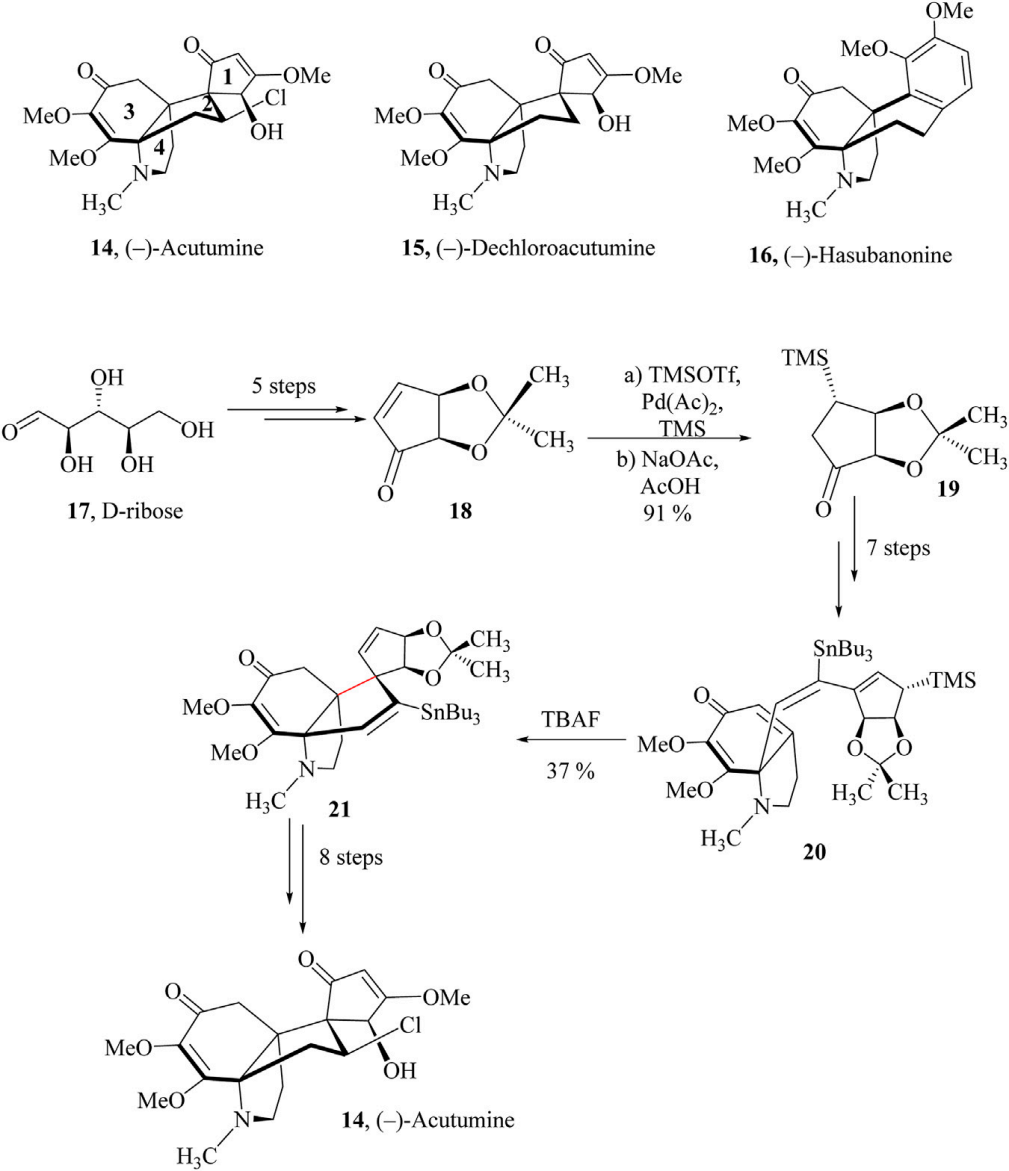
Total synthesis of (−)-Acutumine, **45** (King et al., 2013).

In 2009, Castle and co-workers reported the total synthesis of (−)-Acutumine (**14**) (Li et al., 2009). In the build-up to the synthesis of (−)-Acutumine spirocyclopentenone ring derived from acetonide **17** was prepared in 5 steps from the starting D-ribose, **18** (Scheme 7) (Kim et al., 2020; Smith et al., 2005). Palladium was employed to catalyze the converting of 1,4-disilylation, (Ogoshi et al., 2002), and cleavage of the enoxysilane generated the *β*-dimethylsilyl ketone **19** as a single diastereomer which was taken through seven linear steps to obtain intermediate **20**. HSR was then carried out on **20** using tetrabutylammonium fluoride (TBAF) to induce an intramolecular cyclisation to obtain the key tetracycle **21** as a single diastereomer in 37% yield. The tetracycle **21** was taken through eight other linear steps to yield (−)-Acutumine, **14** (King et al., 2013).

### 2.3 Total synthesis of alotaketals and phorbaketals

**SCHEME 8:**
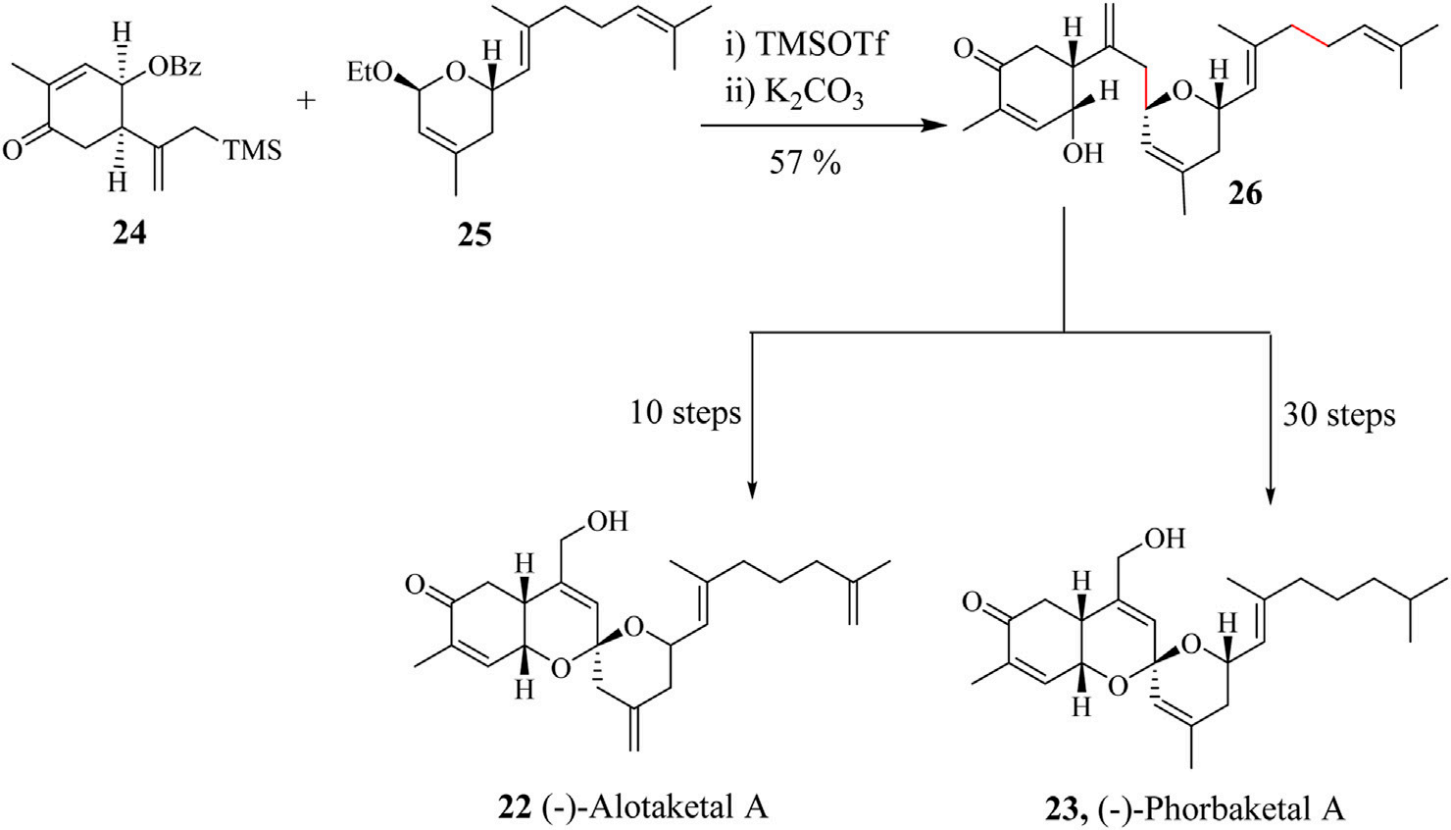
Total synthesis of (−)-Alotaketal A (**22**) and (−)-Phorbaketal A (**23**) involving HSR protocol (Yao et al., 2020).

Alotaketals and Phorbaketals are sesterterpenoid natural products that have been discovered in extracts of marine sponge species (Hubert et al., 2015; Joung et al., 2017; Shirley et al., 2018; Yao et al., 2020). Alotaketal A (**22**) (Scheme 8) was first isolated from a marine sponge *Hamigera sp* in Milne Bay, Papua New Guinea by Kieffer and Andersen in 2009 (Forestieri et al., 2009). To date, five Alotaketals (**A- E**) have been isolated from different other species. Alotaketals share the same tricyclic spiroketal backbone with Phorbaketals (**A- N**) which have had fourteen members isolated and structurally characterised using NMR spectroscopic techniques. Despite their structural similarity, they have different biological activity profiles. Alotaketal A (**22**) was found to increase protein kinase activity and also activate the cyclic adenosine monophosphate (cAMP) cell (*cAMP* is a derivative of adenosine triphosphate, i.e., ATP) signalling pathway in human embryonic kidney cells (HEK293T) (Beavo and Brunton, 2002; Forestieri et al., 2009). Phorbaketal (**23**) on the other hand is cytotoxic against human colorectal, hepatoma, and lung cancer cell lines (Daoust et al., 2013; Lee et al., 2014; Wang et al., 2016; Yao et al., 2020).

Yao et al., used TMSOTf as Lewis acid in catalysing the reaction between moiety **24** and **25** through an HSR to prepare a single diastereomer **26** in 75% yield (Scheme 8) (Yao et al., 2020). Yang completed the synthesis of (−)-Alotaketal A (**22**) in 30 steps with an overall yield of 2.6%. Alotaketals and Phorbaketal, i.e., Spiroketals of this nature have been very challenging to synthesise (Yao et al., 2020). Lee et al., accomplished the synthesis of (−)-Phorbaketal A (**23**) in 10 steps with a yield of 1.04% being the shortest linear sequence steps (Lee et al., 2014).

### 2.4 Synthesis of (+)-Amphidinolide P

Amphidinolides are bioactive natural products isolated from marine dinoflagellates. Kobayashi and co-workers isolated the first amphidinolide A in 1986 (Ishibashi and Kobayashi, 1997; Kobayashi et al., 1986) and other amphidinolides including amphidinolides P **27** from a cultured diniflagellate *Amphidinium sp*. Amphidinolides are characterized by their unique 26-membered macrolide structure have shown antineoplastic activity against cancer cell lines and possess IC_50_ values in the low micromolar range (Heravi and Mohammadkhani, 2018).

**SCHEME 9:**
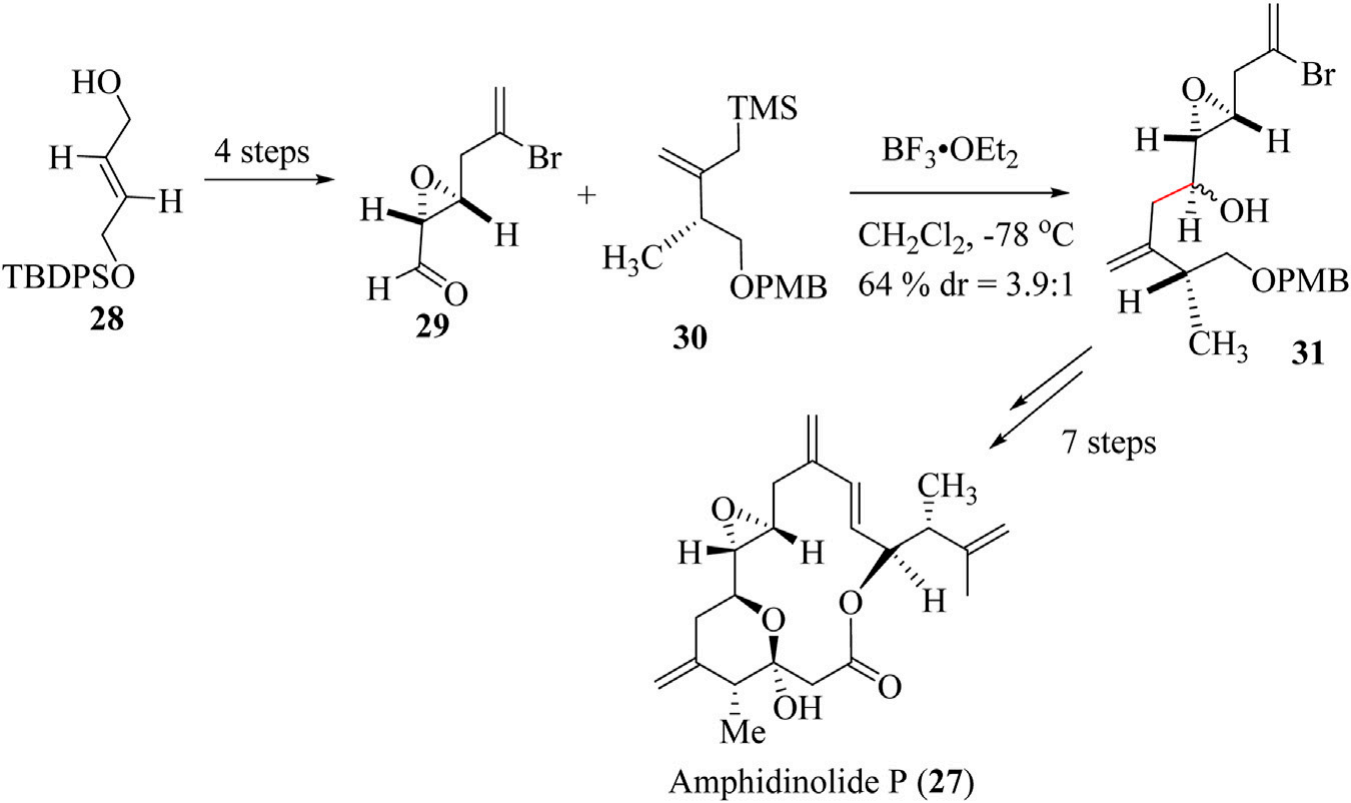
Total synthesis of Amphidinolides P (**27**) (Carreira and Joliton, 2015).

Total synthesis of (−)-amphidinolide P, **27** was reported for the first time in 2000 by Williams and co-workers (Williams and Meyer, 2001). (+)-Amphidinolide P **27** was also prepared from a mixture of chiral precursors **29** and **30** starting from commercially available **28** (Williams et al., 2013). Aldehyde allylation with allylsilane **31** to achieve key intermediate **32** was achieved by adopting the HSR using boron trifluoride etherate as a catalyst Scheme 9 (Carreira and Joliton, 2015; Williams et al., 2013; Williams and Meyer, 2001). Traeatment of intermediate 62 through 7 steps reactions resulted in the formation of the Amphidinolides P (**27**).

### 2.5 Total synthesis of (±)-Aspidospermidine

(±)-Aspidospermidine (**32**) belongs to the Aspidosperma family of alkaloids (Du et al., 2017) which are made up of a pentacyclic ring system and five chiral centres. Members of this family of alkaloids possess antibiotic, anticancer, antimalarial and anti-inflammatory activities. However, the structural complexity of this class of compounds presents a challenge to successful asymmetric synthesis (Du et al., 2017; Ramirez and Garcia-Rubio, 2005).

**SCHEME 10:**
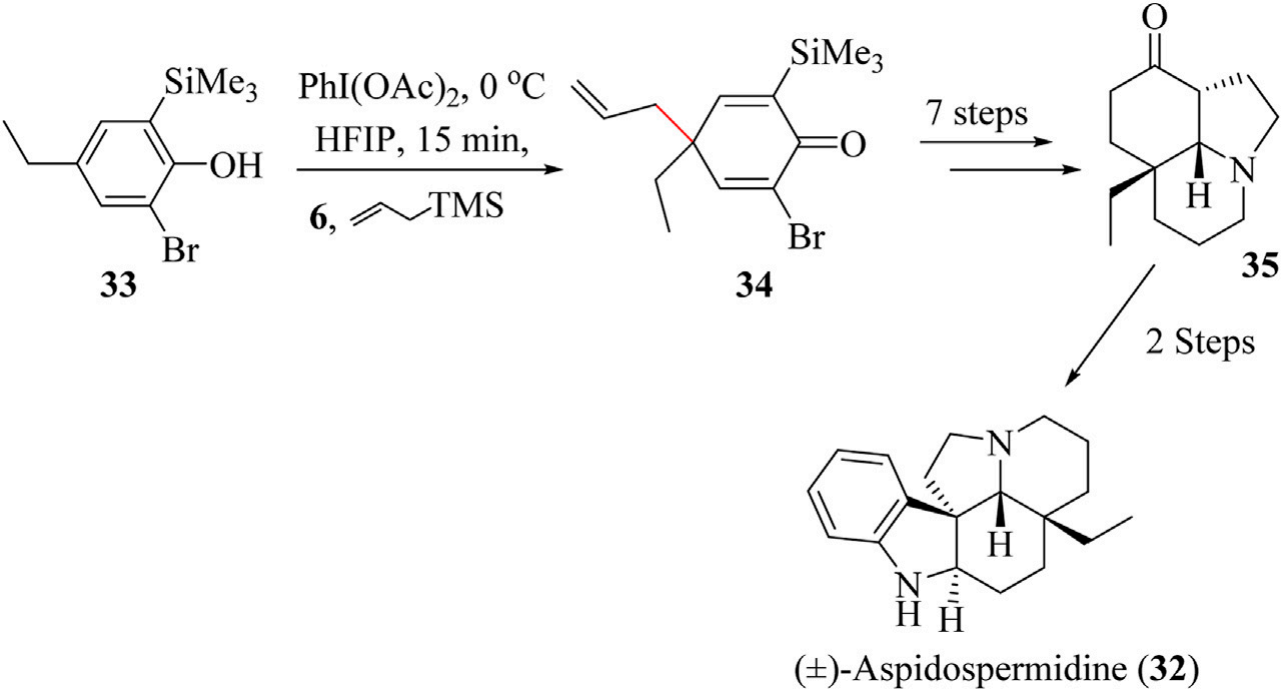
Total synthesis of (±)-Aspidospermidine (**32**) (Stork and Dolfini, 1963).

The key intermediate in the synthetic route of (−)-Aspidospermidine **32** was first prepared by Stork and Dolfini (Stork and Dolfini, 1963). Sabot and co-workers used an alternative approach known as the “aromatic ring umpolung” which starts with polysubstituted phenol **33** which was converted to dienone **34** through HSR using allyltrimethylsilane and trifluoroethanol (TFE) or Hexafluoroisopropanol (HFIP) in the presence of PhI(OAc)_2_ (Scheme 10). The entire total synthesis was completed in 10 steps starting from phenol **33** to give the overall yield of 5.4% (Sabot et al., 2009).

**SCHEME 11:**
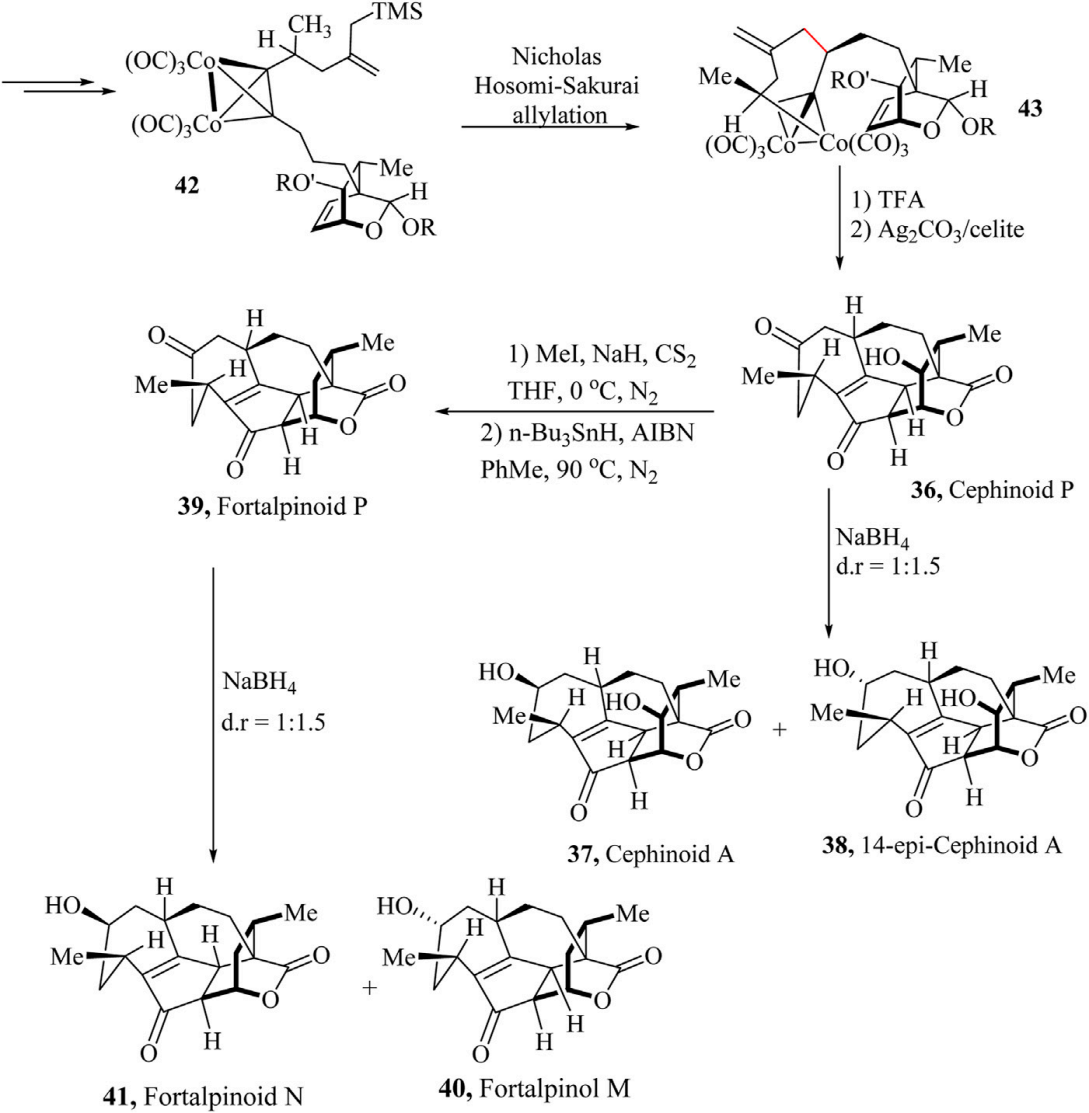
Total asymmetric total synthesis of Cephalotaxus diterpenoids: Cephinoid P, Cephafortoid A, 14-*epi*-cephafortoid A and Fortalpinoids M, N, (Wang et al., 2023).

### 2.6 Total synthesis of Cephalotaxus diterpenoids: Cephinoid P, Cephafortoid A, 14-epi-Cephafortoid A and Fortalpinoids M-N, P

Cephalotaxus diterpenoids are structurally diverse diterpenoids. In 2016, Yue and co-workers isolated this family of compounds from *Cephalotaxus* species (Fan et al., 2017; Shao et al., 2024). This family of natural products showed potential anticancer properties. The intriguing and challenging structure of these compounds was established using X-ray crystallography techniques. Gao and coworkers have recently reported the first asymmetric total synthesis of these natural products and subgroups such as Cephinoid P (**36**), Cephafortoid A (**37**), 14-*Epi-*cephafortoid A (**38**) and Fortalpinoids M (**39**), N (**40**) and P (**41**) from the precursor (S)-trimethyl (4-methyl-2-methylenehex-5-yn-1-yl) silane (Wang et al., 2023).

A universal strategy for the total synthesis of Cephalotaxus diterpenoids bearing unique cycloheptene A rings with chiral methyl groups was adopted. The 7-5-6 tricycle rings of the Cephalotaxus diterpenoids were obtained from the dicobalt hexacarbonyl-propargylic alcohol complex (Scheme 20). An intramolecular Nicholas/Hosomi-Sakurai cascade reaction was developed to form the cycloheptene A-ring bearing a chiral methyl group (Scheme 11). The Pauson-Khand reaction was then used to complete the skeleton of the target molecules. The asymmetric total syntheses of Cephinoid P (**36**), Cephafortoid A (**37**), 14-*Epi-*cephafortoid A (**38**) and Fortalpinoids M (**39**), N (**40**) and P (**41**) were successfully achieved over 15–18 steps from compound **42**. The proposed strategy presents a novel approach for the total synthesis of Cephalotaxus diterpenoids and structurally related akin polycyclic natural products (Wang et al., 2023).

### 2.7 Total Synthesis of Dehaloperophoramidine and (+)-Perophoramidine

Naturally occurring (+)-Perophoramidine **44** was isolated from marine ascidian *Perophora namei* by Ireland and co-workers in the Philippine ascidian organism, *P. namei* (Verbitski et al., 2002). Dehalogenated product Dehaloperophoramidine **44** was obtained from the hydrogenation of (+)-Perophoramidine **45**. The two compounds, **44** and **45** bear a structural resemblance to a complex family of natural products called communesin alkaloids (Communesin F, **46**) (Trost and Osipov, 2015; Wilkie et al., 2016; Zuo and Ma, 2011). They both possess complex chemistry of quaternary chiral centres that makes them synthetically challenging. (+)-Perophoramidine **45** has been reported to possess anticancer activity and this has drawn the synthetic chemist’s attention to them.

**SCHEME 12:**
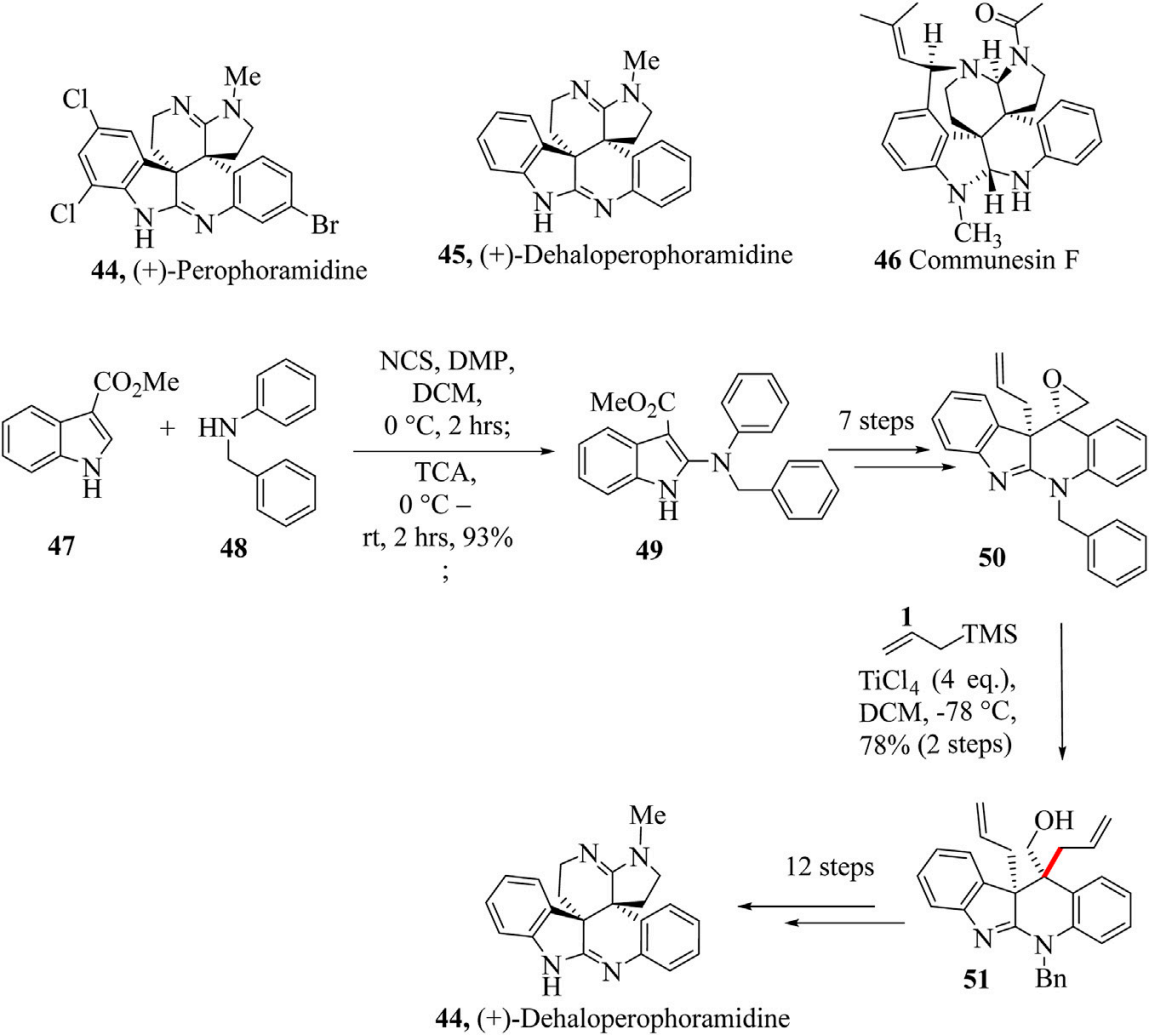
Total synthesis of (+)-Dehaloperophoramidine **51** (Wilkie et al., 2016).

Synthesis of Dehaloperophoramidine **44** started with the reaction of commercially available **47** and **48** to make intermediate **49**, which was taken through seven steps to yield **50**. Key intermediate **51** was treated with allyltrimethylsilane in the presence of TiCl_4_ to give **52** which was taken through 12 extra steps to achieve Dehaloperophoramidine **44** (Scheme 12) (Wilkie et al., 2016).

### 2.8 Total synthesis of deoxopinguisone

Deoxopinguisone (**52**) is a type of Pinguisanes (sesquiterpenes) that occurs naturally in liverworts (Asakawa et al., 2013). Pinguisanes was first isolated and characterised successfully using spectroscopic techniques by Krutov and co-workers in 1972 (Krutov et al., 1973; Uyehara et al., 1985). The distinctive feature of this compound is its spatial configuration of the four methyl group substituents. These methyl groups are specifically oriented in a *β*-configuration in the molecular framework.

Formation of the C4–C9 bond was facilitated by the HSR to give a *cis* stereochemistry between C8 and C9 since the *cis* hindrance system is more stable in solution compared to the *trans*-derivative. The synthesis started with commercially available ketone **53** which was taken through seven sequential steps to obtain intermediate **54** (Supplementary Scheme S9). This intermediate **54** was oxidized with pyridinium dichromate and tert-butylhydroperoxide to obtain the enone intermediate **55** which was subjected to Hosomi-Sakurai cyclisation (HSC) in dry TBAF that led to the concomitant cleavage of the C-Si bond to form intermediate **55**. The HSC of **55** favoured only the formation of the desired *cis* isomer **56** as shown in Supplementary Scheme S9 below (Tori et al., 2001).

From Supplementary Scheme S10, Dthe conformation of the *cis*-isomer shows that the *β-*position from the enone is near the allylic proton that is in the *γ*-position from the TMS group. Also, *cis* -isomer of cyclohexanone is favoured due to steric hindrance (Supplementary Scheme S10) (Tori et al., 2001). The intramolecular cyclisation was accomplished by the HSR in dry TBAF as a catalyst in THF as a solvent to yield 99% compound **56**, but **56** was not formed through the *trans* conformation due to steric hindrance as shown (Supplementary Scheme S10) in the intermediate (Tori et al., 2001). Conversion of intermediate **55** to the final product Deoxopinguisone, **52** was accomplished by Uyehara and coworkers (Uyehara et al., 1985; 1986).

### 2.9 Total synthesis of Ent-Callilongisin B

*Ent*-Callilogisin B (**57**) was isolated from the leaves of *Callicarpa longissima*, along with related compounds, Callilongisin A (**58**), C (**59**), and D (60). *Ent*-Callilongisin B (**59**) is an analogue of 3,4-seco-abietane-type diterpenoid whose structure was established using NMR spectroscopic techniques (Liu et al., 2012). The natural product **57** demonstrated cytotoxic activities against a human prostate cancer cell line. An anti-inflammatory activity of **57** was also observed when tested for superoxide anion production from human neutrophil cells (Kamiya et al., 2021).

**SCHEME 13:**
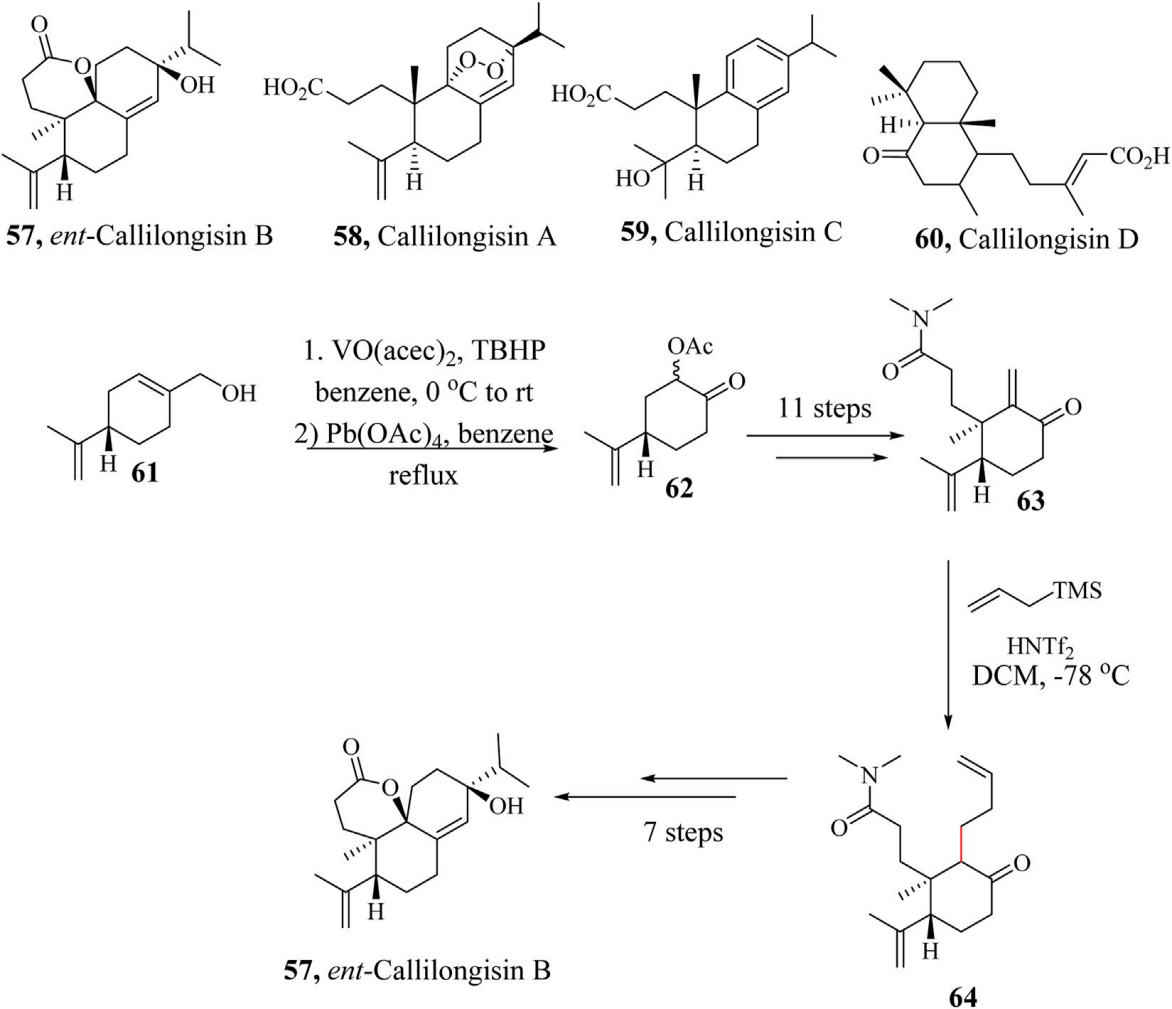
Total synthesis of *Ent*-callilongisin B (**57**) (Kamiya et al., 2021).

In 2021, the first asymmetric total synthesis of tricyclic diterpenoid *Ent*-callilongisin B (**57**) was reported by Kamiya et al. (2021). The synthesis started with the Sharpless oxidation of enantiomerically pure (*S*)-perillyl alcohol **61**, to form acetoxy ketone **62** (Indictor and Brill, 1965; Sharpless and Michaelson, 1973; Tanaka et al., 1974). The synthesis was accomplished following stereo-controlled Michael 1,4-addition and Hosomi−Sakurai allylation of enone **63**, followed by Wacker oxidation, and intramolecular aldol reaction of diketone to construct the six-membered ring and oxidative dearomatisation of phenol intermediate accompanied by diastereoselective δ-lactonisation (Scheme 13). The same research group is also developing enantioselective synthetic methods for other Callilongisin analogues A (**58**), C (**59**) and D (**60**) (Kamiya et al., 2022).

### 2.10 Total synthesis of eribulin mesylate

Eribulin mesylate (**65**) is an anticancer drug made from halichondrin B (**66)**, a natural marine polyether macrolide product (Ortega and Cortés, 2012; Paterson et al., 2011). In 1986, halichondrin B was first isolated from the marine sponge *Halichondrin okadai* by Uemura and co-workers (Kulkarni et al., 2016). Further studies on the structure of halichondrin B led to the discovery of the analogue known as Eribulin mesylate (**65**) with improved anticancer activity against metastatic cancer cell lines. In 1997, Eisai Pharmaceutical Company in collaboration with Kishi et al., reported the total synthesis of the drug in an industrial viable way (Fang et al., 1992; Lee et al., 2016; Li et al., 2018; Stamos et al., 1997).

**SCHEME 14:**
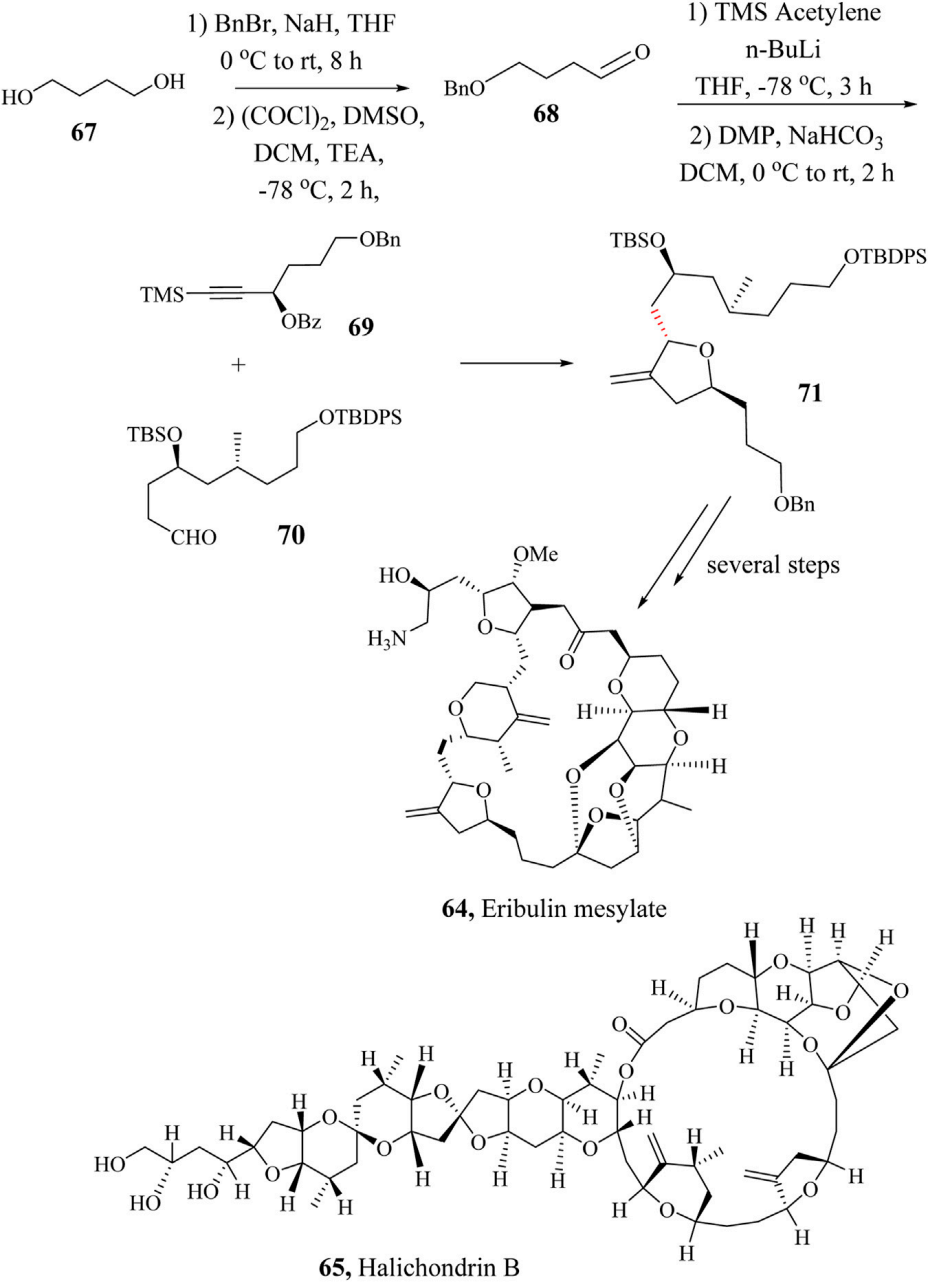
Total synthesis of Eribulin mesylate, **64** (Nasam and Pabbaraja, 2024).

Currently, Pabbaraja et al., reported a more economical, and industrially scalable, approach toward the easy production of the drug (Nasam and Pabbaraja, 2024). The modified stereoselective synthetic approach to the preparation of Eribulin mesylate (**65**) involved the use of commercially available 1,4-butanediol (**67**) to generate key intermediate **68** which was coupled with intermediate **69** in HSR to achieve the key intermediate **70**. The total synthesis of Eribulin mesylate was accomplished by taking the intermediate **70** through several steps (Scheme 14) (Nasam and Pabbaraja, 2024).

### 2.11 Total synthesis of (−)-Galiellalactone

**SCHEME 15:**
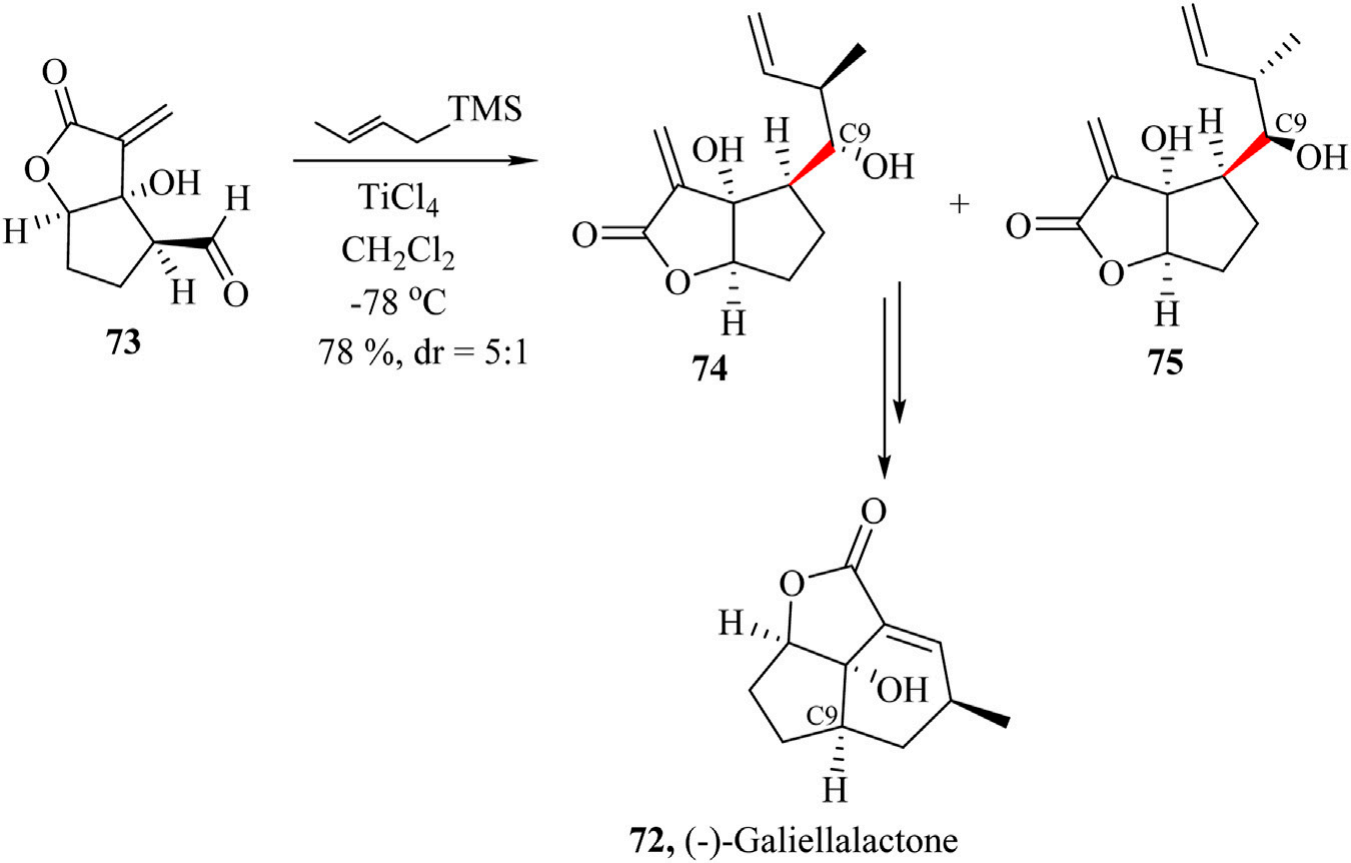
Total synthesis of (−)-Galiellalactone (**72**) (Kim et al., 2015).

(−)-Galiellalactone **72**, (Scheme 15), a fungal metabolite was originally isolated from ascomycete *Galiella rufa* in 1990 (Johansson and Sterner, 2001; Kim et al., 2015). It has been found to possess inhibitory properties against STAT3 (signal transducer and activator of transcription 3) that block the DNA binding of phosphorylated STAT3 without inhibiting phosphorylation and dimerization (Don-Doncow et al., 2014). (−)-Galiellalactone also induces the apoptosis and growth inhibition of human prostate cancer cells. Sterner and co-workers were the first to report the synthesis of the (−)-Galiellalactone and confirmed its stereochemistry unambiguously (Johansson and Sterner, 2001).

Kim et al also used a modified Hosomi−Sakurai crotylation method (Hosomi and Sakurai, 1977; Shing and Li, 1997) to diastereoselectively introduce the C9 side chain with the required terminal alkene for ring closure metathesis. The intermediate **73** was reacted with (*E*)-crotyltrimethylsilane and BF_3_⋅Et_2_O as the promoter to yield compounds **74** and **75** in 88% yield in favour of the undesired **75**. However, when the catalyst was changed to TiCl_4_, the yields dropped to 78% but favoured compound **74** to give a dr of 5:1 (Scheme 15). Further, intermolecular cyclisation of the **75** resulted in the formation of the product with the exact configuration (Kim et al., 2015).

### 2.12 Total syntheses of grayanane diterpenoids: (−)-grayanotoxin III, (+)-Principinol E, and (−)-Rhodomollein XX

Grayanane diterpenoids are naturally occurring secondary metabolites exclusively found in flowering plants of the Ericaceae family (Li et al., 2019) with over 4,000 species (Liu et al., 2024). In addition to grayanane diterpenoids (Cai et al., 2018; Ho and Lin, 1995; Lv et al., 2017; Zhou et al., 2012), Ericaceae plants contain other types of terpenoids, including triterpenoids (Teng et al., 2018; Zhang et al., 2018; Zhao et al., 2018) and meroterpenoids (Huang et al., 2018; Liao et al., 2015; Liao et al., 2017). These compounds feature a distinctive tetracyclic backbone system with an intricate 5/7/6/5 configuration, contributing to their complexity and diversity (Huang et al., 2005; Wang et al., 1998). This family of compounds exhibits various properties, including analgesic, antinociceptive, anticancer, antiviral, antifeedant, insecticidal effects, as well as toxicity and inhibition of protein tyrosine phosphatase 1B (PTP1B) (Li et al., 2019; Liu et al., 2024).

Grayanotoxins were isolated from the leaves of *Leucothoe grayana*, a poisonous shrub native to Japan, between 1930 and 1960 (Kakisawa et al., 1961; Kong et al., 2023) but the stereochemistry at the ring junction was only ambiguously determined in 1970 using X-ray crystallography by Narayanan and coworkers (Narayanan et al., 1970). Grayanotoxins are known to be toxic to both humans and animals, acting by modulating voltage-gated sodium channels. This mechanism accounts for their poisoning effects as well as their analgesic properties. Grayanotoxin III, **76** is the alkaline hydrolysed derivative of Grayanotoxin I, which naturally occurs in *L. grayana* (Kakisawa et al., 1961). Principinol E, **77** was isolated from the aerial parts of *Rhododendron principis* and exhibited significant *in vitro* inhibitory activity against PTP1B with an IC_50_ value of 3.14 ± 0.12 µM (Liu et al., 2014; Z. R; Zhang et al., 2012). Rhodomollein XX, **78** was isolated from fruits of *Rhododendron mole* (Li et al., 2000; Zhou et al., 2014) and exhibited moderate antinociceptive activity (Li et al., 2019).

**SCHEME 16:**
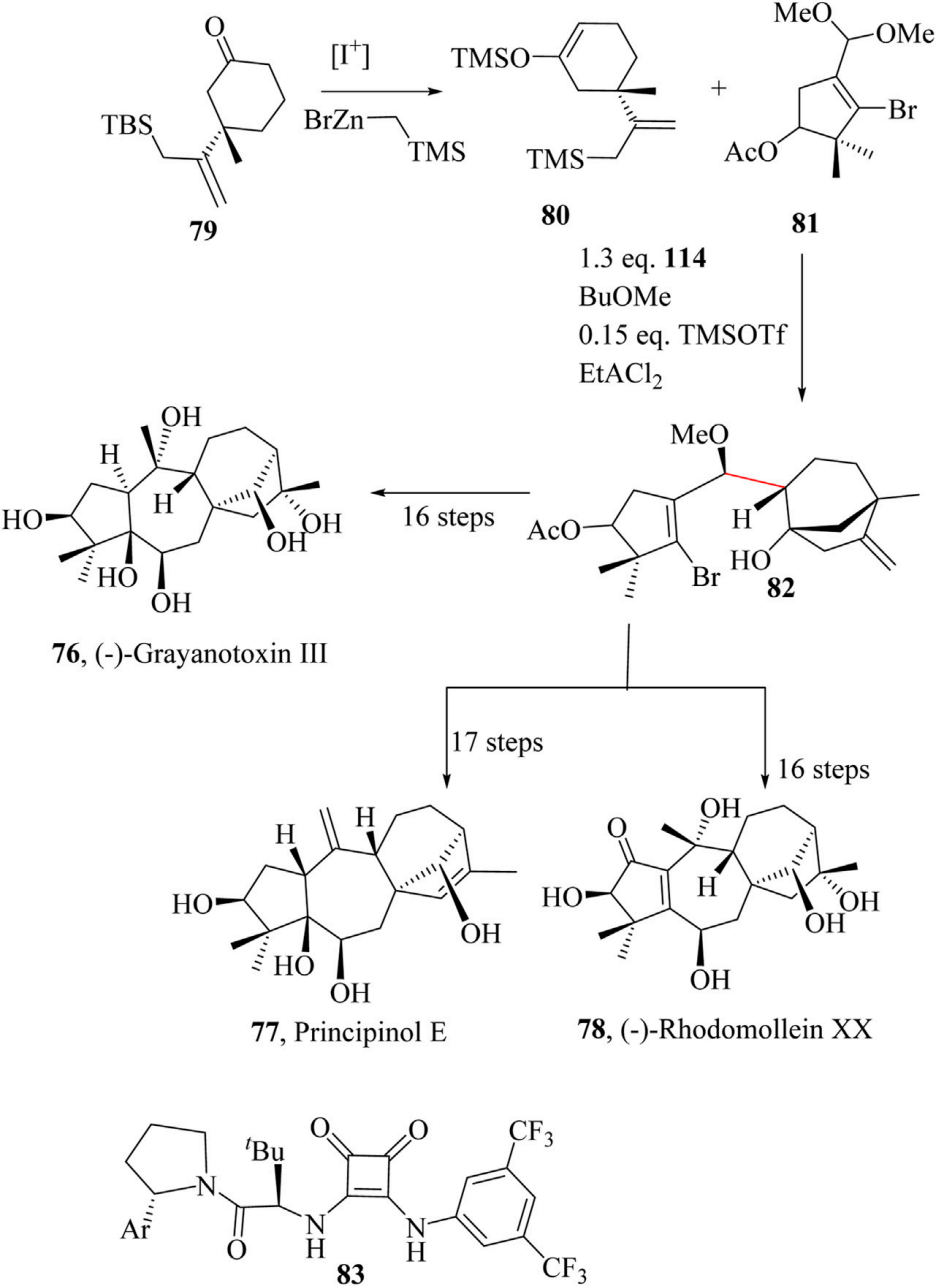
Total syntheses of Grayanane Diterpenoids: (−)-Grayanotoxin III (**76**), (+)-Principinol E (**77**), and (−)-Rhodomollein (**78**) (Kong et al., 2022; 2023).

The total synthesis of **76**, **77**, and **78** began with the synthesis of the key intermediate **80** from **79**. The intermediate **81** was prepared by the asymmetric 1,4-conjugated addition developed by May and co-workers (May et al., 2011) and was treated with TMSOTf in the presence of **83** to foster an intramolecular Mukaiyama aldol reaction followed by the addition of EtAlCl_2_ which mediated the HSR to produce **82** in 62% yield (1.7:1 dr) in a one-pot. A total of 18, 19 or 20 and 18 synthetic steps were followed to obtain (−)-Grayanotoxin III, **76**, (+)-Principinol E, **77** and (−)-Rhodomollein XX, **78** (Scheme 16) (Kong et al., 2022; 2023).

### 2.13 Total synthesis of Heliespirone A and C

Heliespirone A (**84**) is a member of a sesquiterpene group of alkaloids that was isolated from cultivar sunflowers, i.e., *Helianthus annuus L*. by Huang et al. (2011). Heliespirone C (**85**) was isolated in the later part of 2006 and reported with six- and five-membered oxaspirocyclic skeletons, respectively (Huang and Liu, 2010). Heliespirone A (**84**) is anticipated to have a significant impact on the allelopathic action of cultivar sunflowers. Heliespirone C (**85**) demonstrated an inhibitory activity in coleoptile bioassay. Due to their intriguing structural features, biological profiles, and limited availability, these natural products present appealing targets for total synthesis (Huang and Liu, 2010).

**SCHEME 17:**
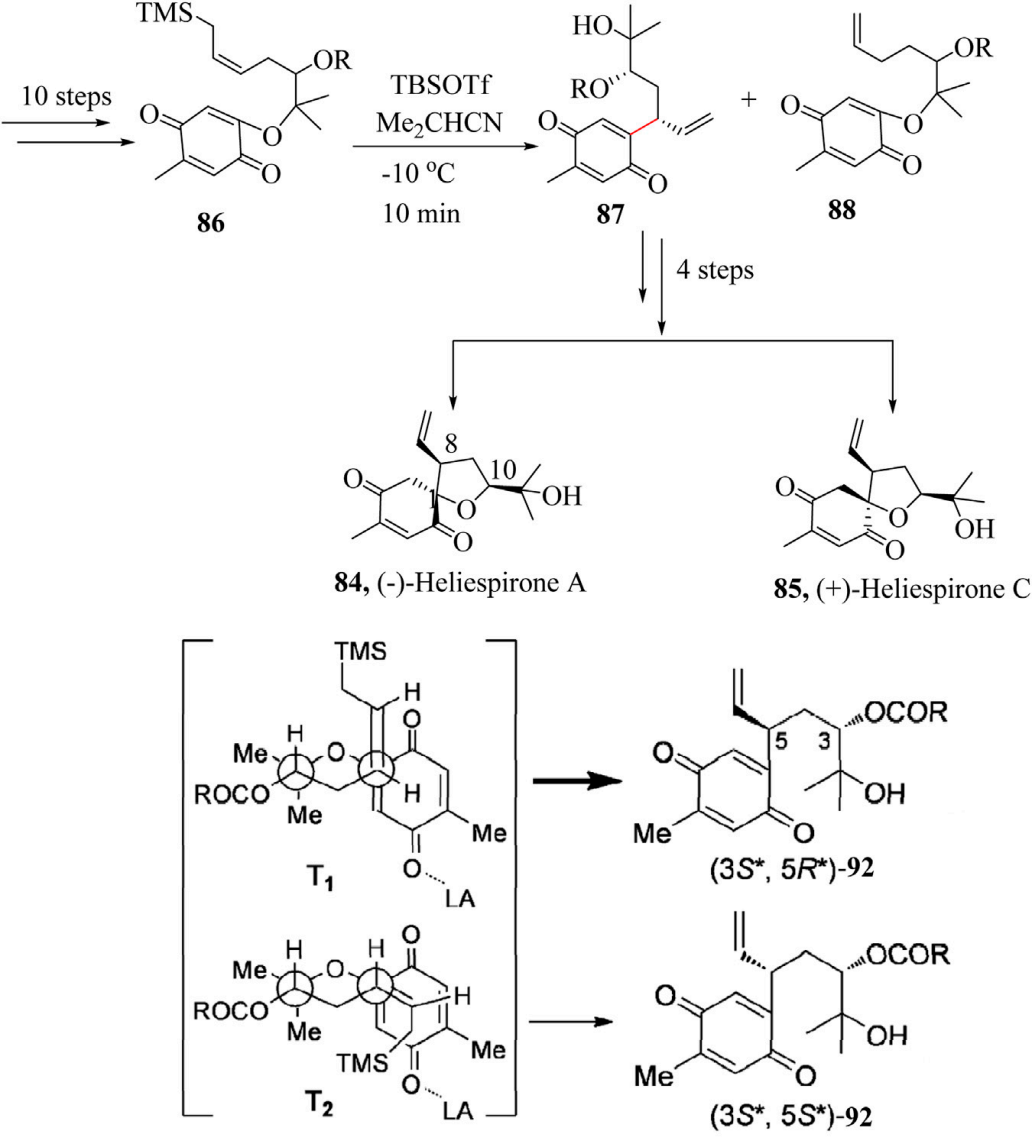
Total synthesis of (−)-Heliespirone A and C (Miyawaki et al., 2012).

Miyawaki et al., in 2012 reported the total synthesis of (−)-Heliespirone A (**84**) and (+)-Heliespirone C (**85**) (Miyawaki et al., 2012). A combination of several synthetic approaches including the HSR was carried out to introduce two stereogenic centers at C-8 and C-10 (Scheme 17). Intramolecular allylation of substrate **86** which had an allylsilane and *p*-benzoquinone substituents was accomplished using *tert*-butyldimethylsilyl trifluoromethanesulfonate (TBSOTf) as a catalyst in isobutyronitrile (Me_2_CHCN) to give a mixture of **87** and **88** in 68% with 8:1 dr. The excellent stereoselectivity in this transformation could be attributed to the preference for transition state **T1** over **T2**. This is due to the stereoelectronically favourable antiperiplanar conformation of **T1**, as well as the steric repulsion between the allylsilane and the benzoquinone groups in **T2** as shown in Scheme 17. Intermediate **88** was taken through 4 linear steps to achieve the two diastereoisomers (±)-Heliespirones A, **84** and C, **85** (Miyawaki et al., 2012).

### 2.14 Total synthesis of Herboxidiene/GEX1A

Herboxidiene *GEX1A* (**89**) a potent phytotoxic polyketide was isolated from *Streptomyces chromofuscus* A7847 by Isaac and co-workers in 1992 (Isaac et al., 1992; Thirupathi and Mohapatra, 2016). Yoshida *et al.* also isolated six polyketide analogues [series (GEX1)] from *Streptomyces sp*, in 2002 (Miyawaki et al., 2012; Sakai et al., 2002; Yun and Panek, 2007). The compound exhibited selective phytotoxicity against a range of broadleaf annual weeds but harmless to wheat (Blakemore, 2002; Sakai et al., 2002). Herboxidiene/*GEX1A* (**89**) is also able to induce GAP1 and GAP2/mitosis (G1 and G2/M) cell cycle arrest in human tumour cell line WI-38 (Ghosh and Li, 2011). It also binds to SAP155, the protein responsible for the pre-mRNA splicing thereby acting as a novel slicing inhibitor (Thirupathi and Mohapatra, 2016).

**SCHEME 18:**
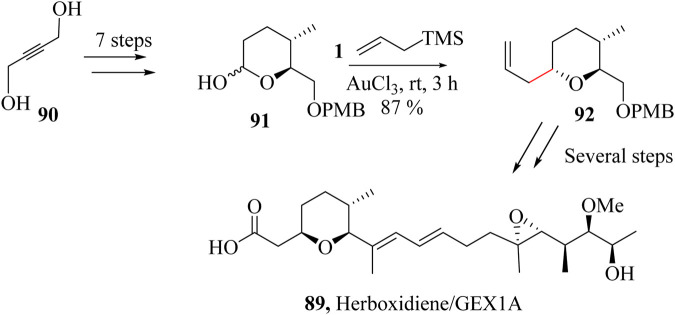
Total synthesis of Herboxidiene *GEX1A,* **89** (Thirupathi and Mohapatra, 2016).

The first total synthesis attempts of herboxidiene*/GEX1A,* **89** was in 1999 by Kocienski and co-workers using direct aldol reaction, Ireland-Claisen rearrangement, and hydroxy-directed epoxidation. The structural complexity of herboxidiene/*GEX1A* (**89**) comprising the presence of nine chiral centres, trisubstituted tetrahydropyran core, and conjugated diene side chain has made its synthesis particularly challenging (Smith et al., 1996). Mohapatra and Thirupathi reported a 22 linear sequence steps toward the synthesis of herboxidiene*/GEX1A* (**89**) (Thirupathi and Mohapatra, 2016). HSR was used to couple lactol **90** which was achieved in 7 steps from 2-butyne-1,4-diol (**91**), with allyltrimethylsilane and AuCl_3_ as a diastereoselective allylation catalyst previously developed for cyclic hemiacetals as shown in Scheme 18. Compound **92** was the key intermediate needed to synthesize Herboxidiene **89** (Thirupathi and Mohapatra, 2016).

### 2.15 Total synthesis of Huperserratines A and B

*Lycopodium* alkaloids are a large group of natural products with unique heterocyclic frameworks found in the Lycopodiaceae family. Following the isolation of lycopodine by Bödeker (1881), Harrison et al. (1961), Wada et al. (2019), more than 300 *Lycopodium* alkaloids have been isolated, and their structures established using various spectroscopic techniques including X-ray crystallography (Ma and Gang, 2004; Siengalewicz et al., 2013). These compounds exhibit distinguishable polyfused-bridged structures and demonstrate remarkable biological activities. For instance, Huperzine A, a lycodine-type lycopodium alkaloid isolated from *Huperzia serrata*, is a highly specific and potent inhibitor of AChE (Xing et al., 2014). It is also found to be effective in the treatment of Alzheimer’s disease (AD) in China, and used as a dietary supplement in the United States (Liu et al., 1986; Xu et al., 1999). In 2020, ZhaO and co-workers isolated two *Lycopodium* alkaloids Huperserratines A (**93**) and B (**94**), from *H. serrata* (Wu et al., 2020). The absolute configurations of these compounds were determined using single-crystal X-ray diffraction. The compounds were the first reported macrocyclic lycopodium alkaloids with a 5-azabicyclo [10.4.0]hexadecane structure. They were also the second and third examples of lycopodium alkaloids containing an oxime group in the molecule. In addition, Huperserratine A (**93**) showed moderate anti-HIV-1 activity with an EC_50_ value of 52.91 μg mL^−1^ (Deng et al., 2022).

**SCHEME 19:**
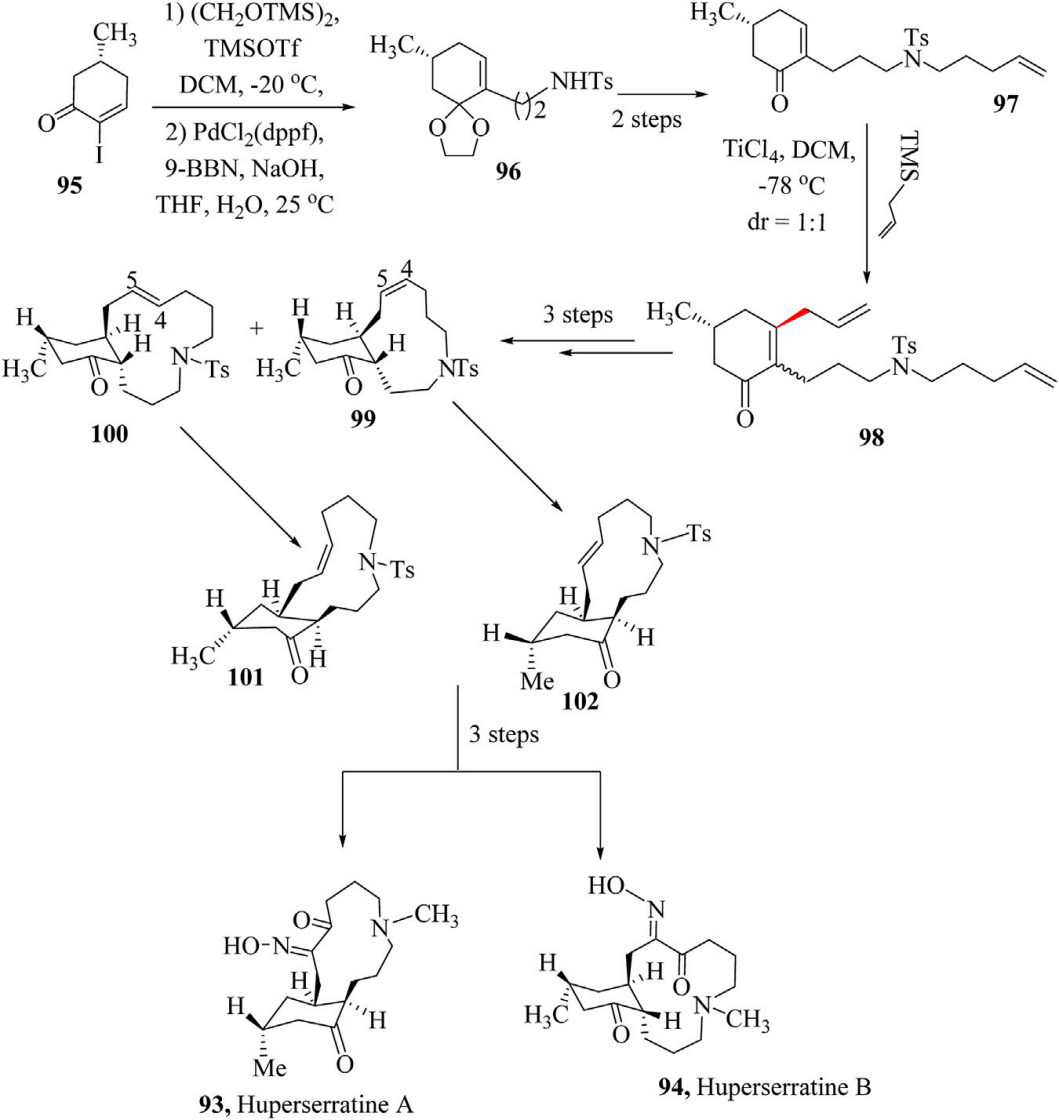
Total synthesis of Huperserratine A (**93**) and B (**94**) (Deng et al., 2022).

ZhaO and co-workers reported the first total synthesis of Huperserratines A (**93**) and B (**94**) through a 12-step sequence including Suzuki-Miyaura coupling, the HSR, the ring-closing metathesis, the dihydroxylation, and Swern oxidation (Scheme 19) (Zhao et al., 2021). The synthesis of Huperserratines A (**93**) and B (**94**) started with (*5R*)-2-iodo-5-methyl-2-cyclohexen-1-one (**95**), which was transformed into the intermediate, **96** by a Pd-catalysed Suzuki–Miyaura reaction followed by modification through *N*-alkylation to obtain compound **97**. The 1,4-addition was completed using a TiCl_4_-catalyzed HSR of enone **98** with allyltrimethylsilane, yielding 3,5-*trans*-cyclohexanone **99**. Epimerization at the α-position of the ketone occurred under these conditions, resulting in a 1:1 mixture of diastereomers. After three steps, diastereomers **101** and **102** were produced, respectively. After the preparation of the oxime followed by deprotection to give Huperserratines A (**93**) and B (**94**) (Deng et al., 2022; Wu et al., 2020).

### 2.16 Total syntheses of (±)-Isonitramine, (−)-Sibirine, and (+)-Nitramine

(±)-Isonitramine (**103**), (−)-Sibirine (**104)**, and (+)-Nitramine (**105**), are spiropiperidine class of alkaloids possessing chiral quaternary carbon centres on their 2-azaspiro [5,5]undecane-7-ol core and were all isolated from *Nitraria sibrica* and *N. Schoberi* (Manske, 1960; Pandey et al., 2011). The three compounds demonstrated good biological activities against the proliferation of cancer cell lines (Boubaker et al., 2011).

**SCHEME 20:**
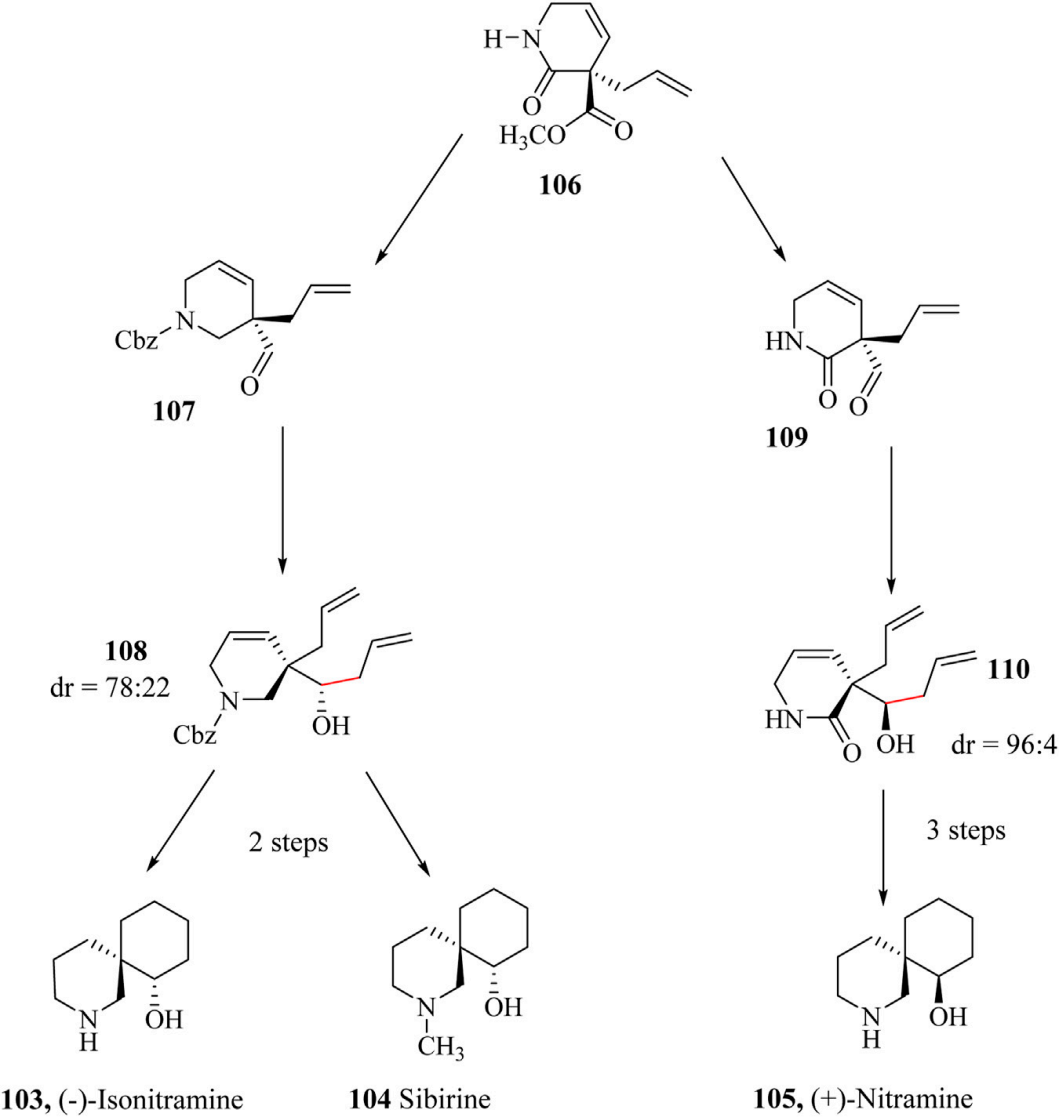
Application of HSR total synthesis of (−)-Isonitramine (**103**), (−)-Sibirine (**104**), and (+)-Nitramine (**105**) (Pandey et al., 2011).

The total synthesis of these alkaloids has been reported although targeted products are obtained as racemic mixtures (Westermann et al., 1993). These molecules appear simple but are challenging to synthesise due to their contiguous chiral centres. The total synthesis of (−)-Isonitramine (**103**) and (−)-Sibirine (**104**) started with the pure enantiomer **106** (Scheme 20). The ester group on **107** was reduced to an alcohol using LiAlH_4_ followed by protection of the amine group using benzyl chloroformate in dioxane/water and subsequent oxidation to yield intermediate **108**. An allylation of intermediate **108** with allyltrimethylsilane in the presence of BF_3_⋅Et_2_O was accomplished via the HSR to give the key intermediate **109** in 92% with 78:22 diastereoselectivity. Synthesis of (+)-Nitramine (**105**) started with the conversion of the ester group of enantiopure **106** to aldehyde **109** which was treated with allyltrimethylsilane in the presence of SnCl_4_ to yield intermediate **110** in 87% with diastereoselectivity of 96:4. The intermediates **108** and **110** were each taken through 2 and 3 steps independently to yield 42%, 38%, and 25% for (−)-Isonitramine (**103**), (−)-Sibirine (**104**), and (+)-Nitramine (**105**), respectively (Pandey et al., 2011; Yang et al., 2021).

### 2.17 Total synthesis of (−)-Kumausallene

(−)-Kumausallene, **111** is sesquiterpene secondary metabolite belonging to a family of non-isoprenoids with a unique bromoallene or enyne moiety and also containing a dioxa-bicyclo [3.3.0] octane core (Das and Ramana, 2015; Hoffmann-Röder and Krause, 2004). Kurosawa *et* al first reported the isolation of (−)-Kumausallene, **111** from red algae *Laurencia Nipponica* Yamada collected at Kumausu, near Otaru, Hokkaido, Japan (Evans et al., 1999; Suzuki et al., 1983). Other members of the enyne containing compounds with dioxa-bicyclo [3.3.0] octane core shown in Supplementary Figure S2 include (+)-Panacene, **112** (Boukouvalas et al., 2006; Feldman, 1982; Feldman et al., 1982) and (−)-Aplysiallene, **113** (Wang and Pagenkopf, 2007); the eight- and nine-membered cyclic ethers (+)-Laurallene, **114** (Crimmins et al., 2010; Saitoh et al., 2003), (+)-Isolaurallene, **115** (Crimmins et al., 2002; Crimmins and Emmitte, 2001) (+)-Itomanallene A, **116** (Jeong et al., 2010) and (+)-Microcladallene B, **117** (Park et al., 2007).

**SCHEME 21:**
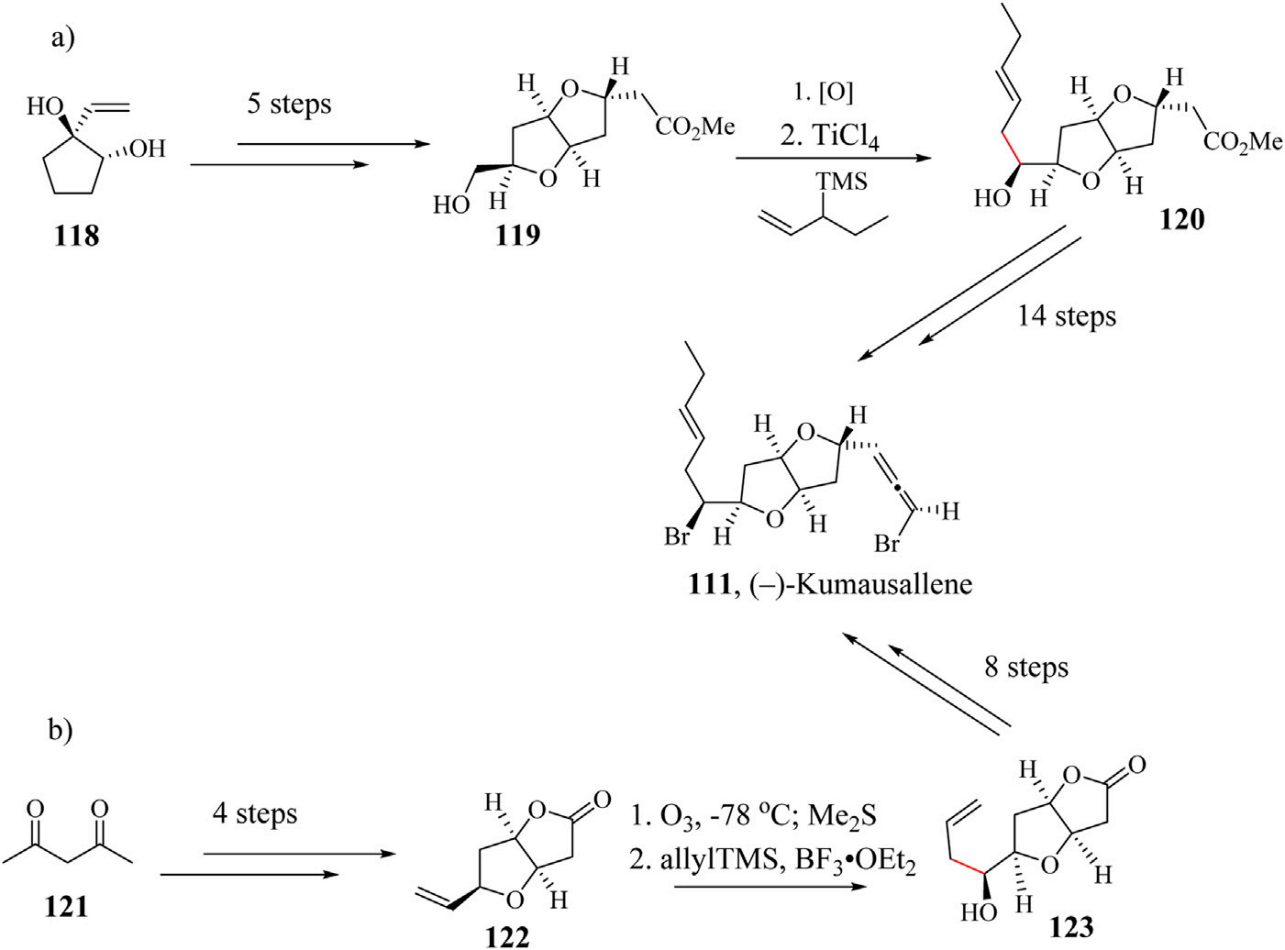
Total synthesis of Kumausallene reported by **(a)** Werness et al. (2013) and **(b)** Werness and Tang (2011).

The first total synthesis of (±)-Kumausallene (**111**) was reported by Overman and his group using a ring annulation strategy (Brown et al., 1991; Grese et al., 1993). It has also been enantioselectively synthesized by Evans et al., using an acyl radical cyclization to construct the tetrahydrofuran ring followed by an efficient biomimetic strategy (Evans et al., 1999). Several other successful syntheses of (±)-Kumausallene (**111**) has been reported in literature (Grese et al., 1993; Werness and Tang, 2011). In their 20 steps synthetic attempt, Werness et al oxidized hydroxyl ester intermediate **119** which was synthesized through 5 steps starting from compound **118** to the corresponding aldehyde and subsequently installed the pentenyl side chain through (Werness et al., 2013; Werness and Tang, 2011). The final product (−)-Kumausallene was achieved after 14 sequential steps as shown in Scheme 21a. Tang *et al.*, used BF_3_⋅OEt_2_ as a Lewis acid in a HSR after the ozonolysis of compound **122** which was synthesized in four steps from acetylacetone **151** to generate pentene linker with the right stereochemistry (Scheme 21b). The HSR proceeded with a diastereoselectivity of a 4:1 ratio favoring the desired stereoisomer. Compound **123** was taken through 8 extra steps to give the title compound in 5.4% yield (Werness and Tang, 2011).

### 2.18 Total synthesis of Leiodermatolide A

Leiodermatolide A, **124** is a 16-membered polyketide macrolide skeleton, featuring an unsaturated side chain terminating in a δ-lactone. It was isolated in 2008 from marine invertebrate sponge lithistid *Leiodermatium Sp*. in Florida (Paterson et al., 2011). It possessed antimitotic selective cytotoxicity towards the pancreatic cancer cell lines AsPC-1, PANC-1, BxPC-3, and MIA PaCa-2, and potent cytotoxicity against skin, breast and colon cancer cell lines (Guzmán et al., 2016; Siu et al., 2021).

**SCHEME 22:**
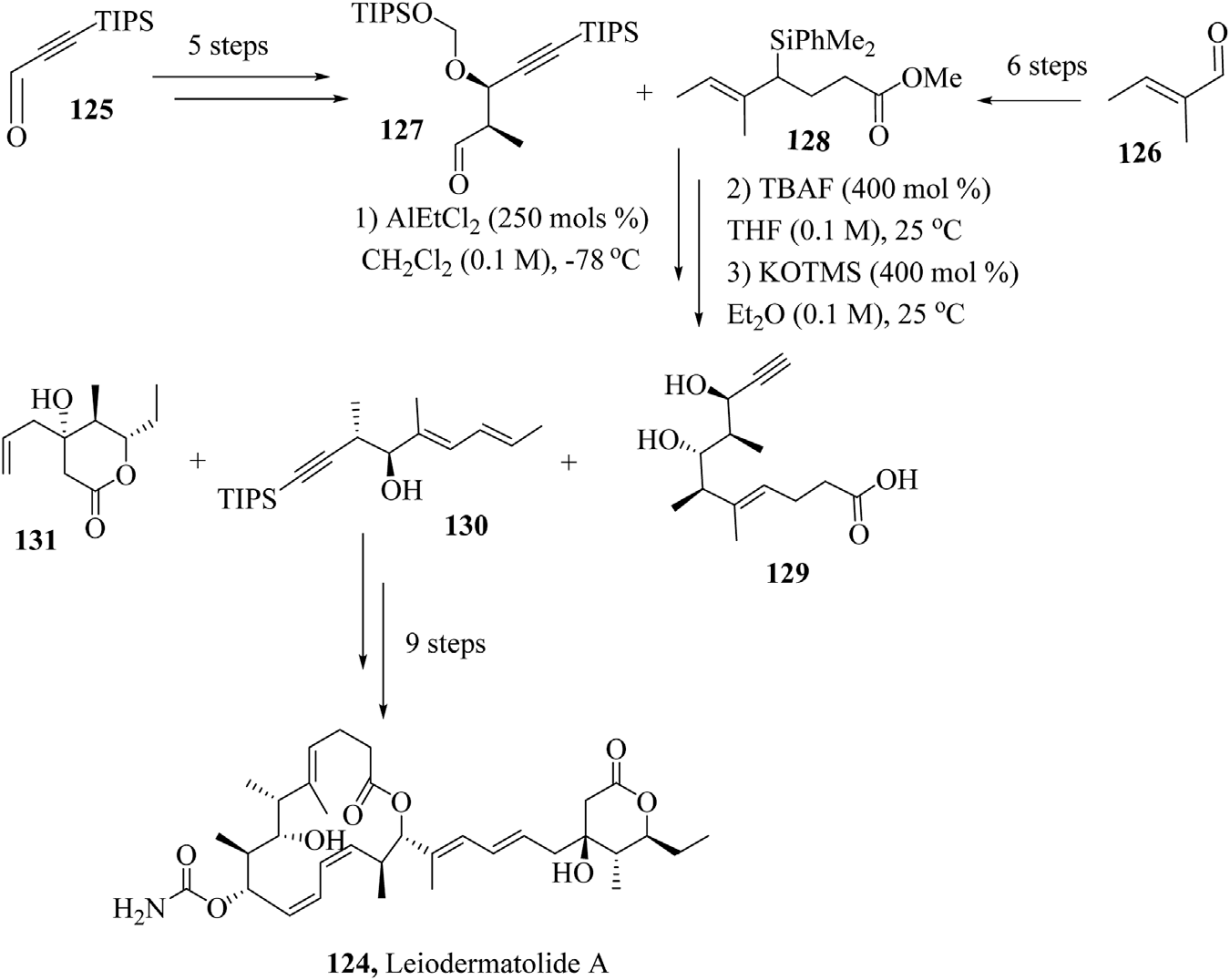
Total synthesis of Leiodermatolide A, **124** (Siu et al., 2021).

Krische and coworkers reported a 13-step total synthesis of leiodermatolide A, by reacting together fragments **124**, **125** and **126** synthesised independently from commercially available starting materials (Scheme 22). Intermediates **127** and **128** were synthesized from starting materials **125** and **126** independently in 5 and 6 steps respectively. Aldehyde **127** and allyl silane **158** were reacted in a chelation-controlled procedure using AlEtCl_2_ followed by TBAF mediated HSR and exhaustive deprotection of the product to yield fragment **129**. Fragments **129, 130** and **131** were then combined sequentially to yield Leiodermatolide A, **124** (Siu et al., 2021).

### 2.19 Total synthesis of Lycopodine

Lycopodine, **132** belongs to a family of over 300 naturally occurring *Lycopodium* alkaloids with a diverse structural backbone (Harrison et al., 1961). It was first isolated in 1881 by Ayer *et al* (Harrison et al., 1961; Yang and Carter, 2010) but its structure was not elucidated until 1960 by Harrison and coworkers (Ayer and Cruz, 1993). Some of the members of this family of compounds possess medicinal properties that can reversibly inhibit acetylcholinesterase and increase learning and memory efficiency (Liu et al., 1986). Lycopodine is made up of four haxacyclic rings fused with five asymmetric centres like lycodine (Liu et al., 1986).

**SCHEME 23:**
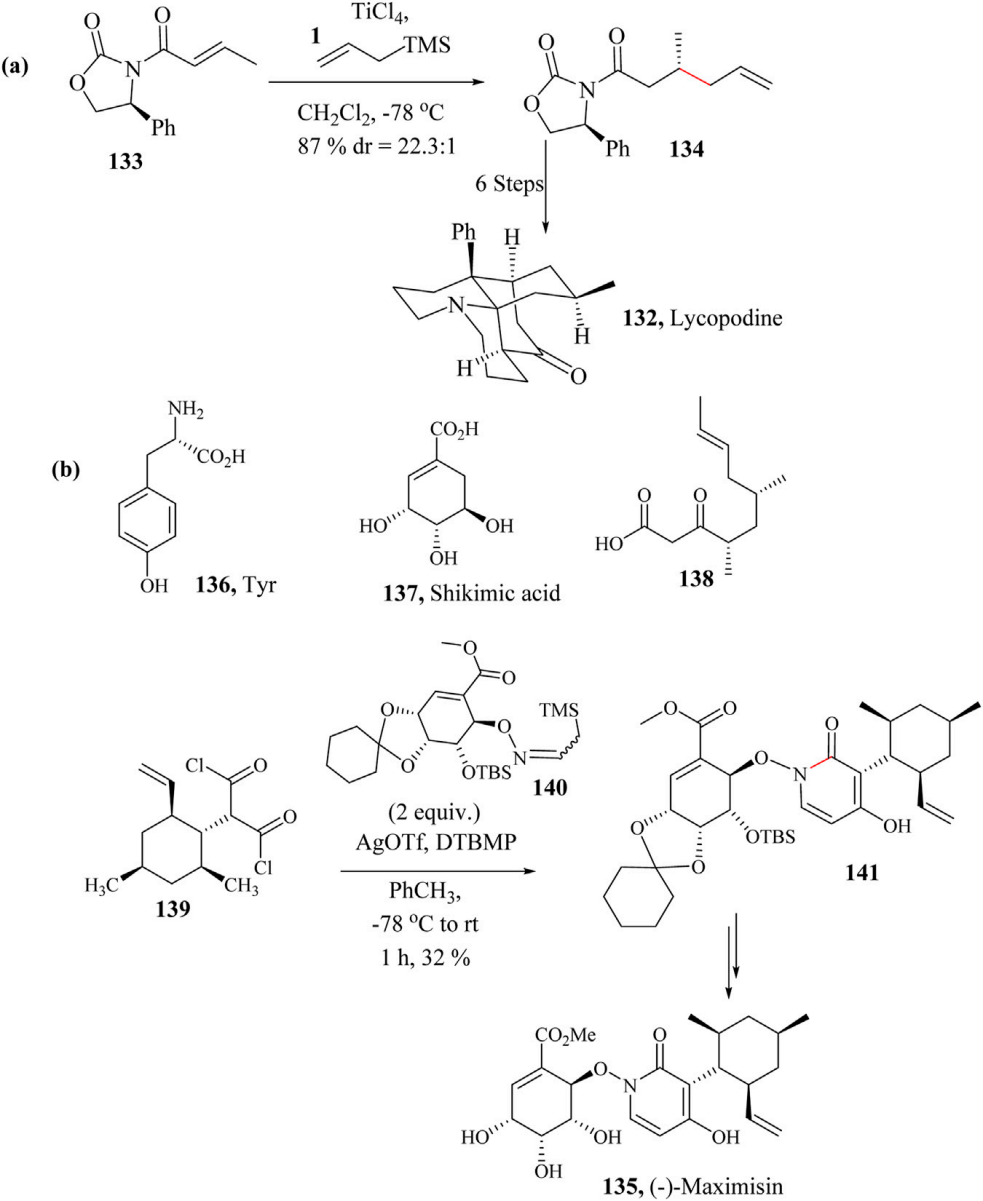
Total syntheses of **(a)** Lycopodine (**132)** and **(b)** (−)-Maximiscin (**135**) involving HSR step (Mcclymont et al., 2020; Wada et al., 2019).

Several total synthetic techniques have been reported for the preparation of natural **132**. Asymmetric synthesis was first reported by Yang and Carter (2010), Ma et al. (2016). Wada and co-workers also reported a concise asymmetric total synthesis of **162** by applying the HSR in the first step (Scheme 23a). Seven synthetic pathways were followed in the total synthesis of Lycopodine which started with the conversion of the commercially available crotonamide **133** to diastereoselectively make **134** (22.3: 1 dr) as shown in Scheme 23a (Wada et al., 2019).

### 2.20 Total synthesis of (−)-Maximiscin

(−)-Maximiscin **135** is an alkaloid made from three different metabolic pathways that incorporates a central 1,4-dihydroxy-2-pyridone derived from tyrosine 137 (Scheme 23b), attached to the ester of shikimic acid **137** and cyclohexyl of a polyketide **141** (Schmidt et al., 2003). 4-Hydroxy-2-pyridone family of alkaloids (Du et al., 2014) to which (−)-Maximiscin belongs are known to be unstable and usually fragment in isolation but possess interesting biological activities (Schmidt et al., 2003). (−)-Maximiscin **135** is a fungal metabolite isolated from the fungus *Tolypocladium* which demonstrated tumor suppression activity in animal models (Du et al., 2013; Du, et al., 2014).

The total synthesis of (−)-Maximiscin **135** faced challenges due to the instability of its structure. Specifically, shikimate and pyridone residues tend to fragment when synthesised. Mcclymont and co-workers completed the total synthesis of Maximiscin **135** by following a modified enantioselective HSR using AgOTf as a promoter to activate the diacid chloride **139** as an electrophile in the presence of **140** and use the *β*-silicon effect of the Si−C to enhance the nucleophilicity of the nitrogen (Déléris et al., 1980; Mcclymont et al., 2020) (Scheme 23b).

### 2.21 Total synthesis of (+)-Ophiobolin A

**SCHEME 24:**
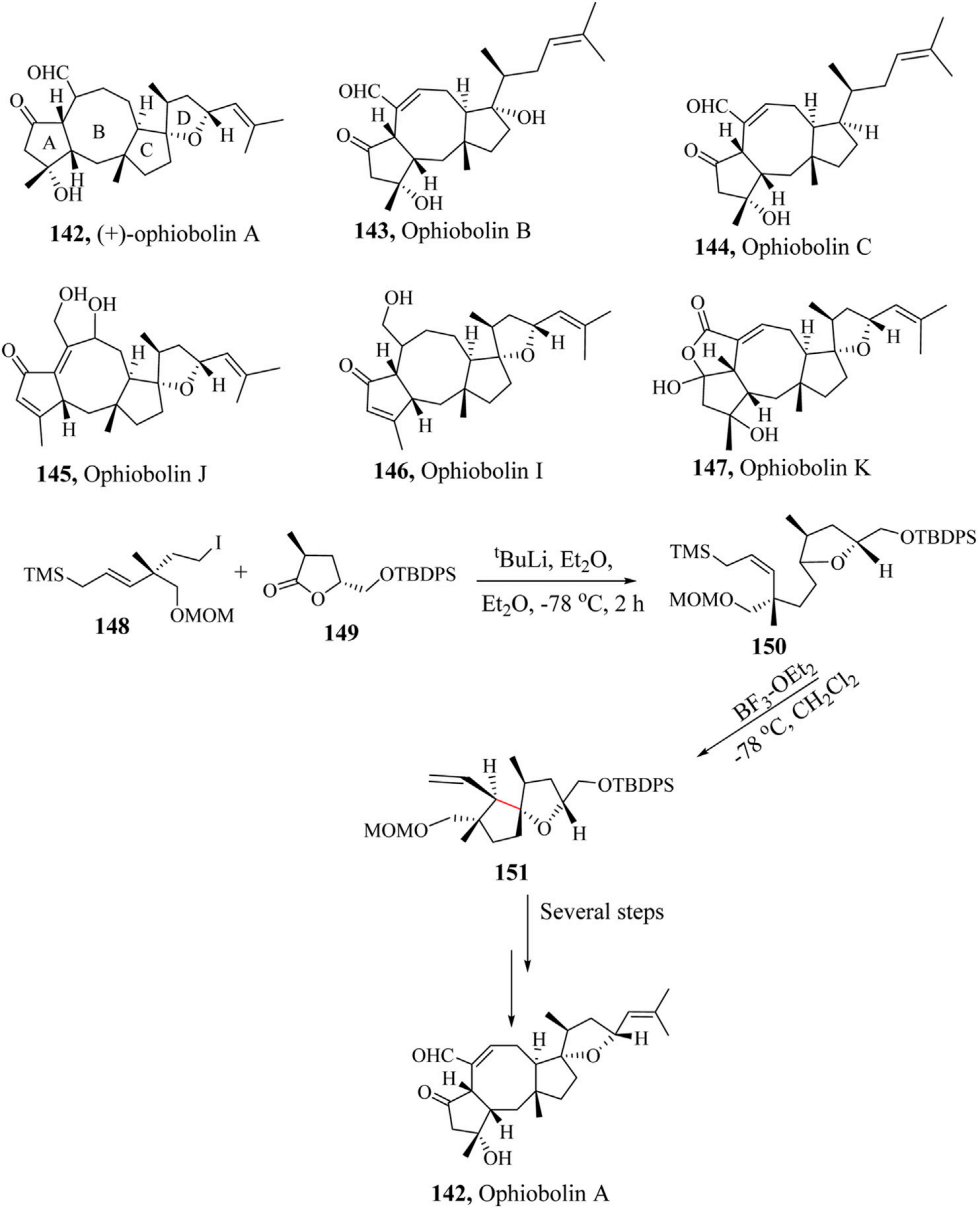
Application of HSR in total synthesis of Ophiobolin A, (**142**) (Tsuna et al., 2013).

Ophiobolins A-K (**142–147**) are terpenoids with 5,8,5-membered carbocyclic ring systems (Scheme 24). The complexity of the structure and intriguing biological activities of Ophiobolins A-K have attracted the attention of the organic synthetic community. (+)-Ophiobolin A, **142** has a unique 5,8,5,5 tetracyclic ring system and eight chiral centres isolated from *Ophiobolus miyabeanus* in 1958 by Ishibashi and co-workers. Its structure was fully established in 1965 by Nozoe et al. (1965), Rowley et al. (1989). Biological screening of **172** has revealed that it induces apoptotic cell death in the L1210 cell-lines, (Fujiwara et al., 2000), inhibits calmodulin-activated cyclic nucleotide phosphodiesterase, and also shows cytotoxicity to cancer cell lines A-549, Mel-20, and P-335 (IC_50_ values that range from 62.5 to 125 µM) (Li et al., 2013).

A number of synthetic attempts have been made toward the total synthesis of Ophiobolin by Kishi and co-workers (Li et al., 2013). Tsuna et al., reported the first total synthesis of (+)-ophiobolin A (**142**) in 2013. A protected *tert*-butyldiphenylsilyl (TBDPS) intermediate **148** was reacted with the methoxymethyl (MOM) intermediate **149** to generate the complex intermediate **150** which was taken through an intramolecular HSR to form the key intermediate spirocyclic compound **151** in 45% yield using BF_3_⋅OEt_2_ catalytic system. Further transformation of the key intermediate result in the production of the targeted product **152** as illustrated in Scheme 24 (Tsuna et al., 2013).

### 2.22 Total synthesis of (+)-Paniculatine

(+)-Paniculatine **152** belongs to the Lycopodium family of alkaloids and was first isolated by Castillo and his group in 1975 (Castillo et al., 1975; 2011). Other members of the Lycopodium family such as (−)-Magellanine **153** (Loyola et al., 1979) and (+)-Magellaninone **154** (Maclean, 1985) were also isolated from *Lycopodium paniculatum* by Casillo and co-workers. (+)-Paniculatine **152** has a complicated 6-5-5-6-fused tetracyclic diquinane core structure made up of seven chiral centres woven in a tetracyclic framework (Ma and Gang, 2004). *Lycopodium* family possesses anti-inflammatory activity and acetylcholinesterase inhibitory properties and are being studied as potential drugs for the treatment of Alzheimer’s and other neurodegenerative diseases (Ma and Gang, 2004; Saito et al., 2023).

The first total synthesis of (+)-Paniculatine (**152**) was reported by Sha and co-workers in 1999 using tandem cyclisation reactions that involve HSR (Sha et al., 1999). Mukai and co-workers, stereospecifically synthesized (+)-Paniculatine **152**, (−)-Magellanine **153**, (+)-Magellaninone **154** (Supplementary Scheme S12) in 43–45 unprecedented synthetic steps in 2007 (Kozaka et al., 2007). Yan et al. proposed a concise palladium-catalyzed reaction in 2014, leading to the synthesis of (+)-Paniculatine, (−)-Magellanine, and (+)-Magellaninone in 12 steps (Chen et al., 2018; Liu et al., 2019). Liu et al., developed a more concise synthetic route for the synthesis of (+)-Paniculatine, achieving the synthesis in 10 steps. This involved the HSR of compound **155** to yield the desired intermediate **156** with a high diastereoselective ratio (dr > 20:1) at −78°C using TiCl_4_ as depicted in Supplementary Scheme S12. The overall yeild was 12%. The authors proposed that the same reaction protocol could be used to synthesise (−)-Magellanine **155** and (+)-Magellaninone **154** as well (Liu et al., 2019).

### 2.23 Total synthesis of (+)-Penostatin E

Penostatin E **157** was isolated from a marine-based alga *Enteromorpha intestinalis*, a strain of *Penicillium sp.* (Jansma and Hoye, 2012). The biological activity of this compound attracts much interest from the organic synthetic community. When screened against cancer cell lines (P388 leukaemia cell lines), an ED_50_ value of 0.9 μg/mL was obtained, making it a potential anticancer agent.

**SCHEME 25:**
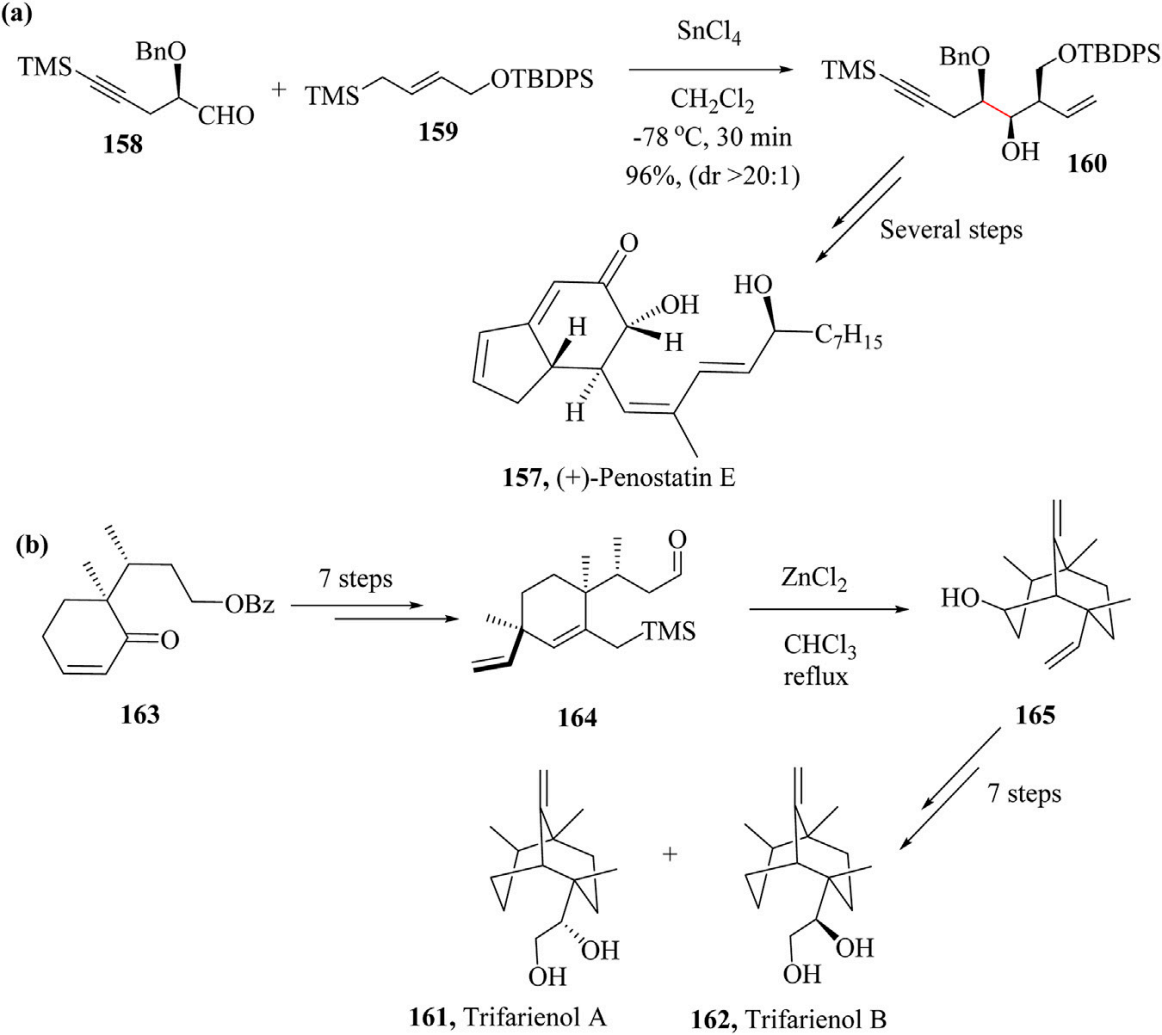
Total syntheses of **(a)** (+)-Penostatin E, **(157)** and **(b)** Trifarienols A **(161)** and B **(162)** showing applications of HSR step (Fujioka et al., 2014; Takahashi et al., 2009).

Fujioka et al reported the first successful synthesis of (+)-Penostatin E, **157** following the HSR method to prepare enyne **150** from aldehyde **158** and allysilane **159** which were both synthesized from (*R*)-glycidyl isobutyrate and (*E*)-4-(trimethylsilyl)but-2-en-1-ol, respectively (Scheme 25a). Enyne **160** was diastereoselectively obtained in 96% yield after reacting **158** and **159** in the presence of SnCl_4_ without loss of enantiopurity. The synthetic pathway was simple and could be employed in the synthesis of other Penostatin derivatives. From compound **160** to the title compound **157** took several steps (Fujioka et al., 2014; Jansma and Hoye, 2012).

### 2.24 Total synthesis of Trifarienols A and B

Trifarienols A (**161**), and B (**162**) are sesquiterpenes that were isolated from *Cheilolejeunea trifaria,* a liverwort of Malaysian origin with a trifarane carbon backbone (Hashimoto et al., 1994; Huang and Forsyth, 1995). These compounds **161** and **162** are structurally complicated biologically active sesquiterpenes that exhibit anticancer, antifungal, and insect antifeedant properties. The complexity of this class of compounds is hidden in the highly substituted bicyclo [3.3.1] nonane moiety and the *exo*-methylene group. The bicyclo [3.3.1] nonane backbone of Trifarienols A (**161**) and B (**162**) is also found in some naturally occurring compounds such as hyperforin, aristophenones, guttiferones, garsubellin A, papuaforin A, and upial.

The first successful Trifarienols A (**161**) and B (**162**) synthesis was by Huang and co-workers (Huang and Forsyth, 1995) in 16 steps with an overall enantiomeric yield of 3%. Tori and co-workers attempted enantioselective synthesis of Trifarienols A (**161**) and B (**162**) in 1999 where they used (2*RS*,3*R*)-2,3-dimethylcyclohexanone as starting material and had an overall yield of 1.8% and 1.4% respectively (Tori et al., 1999). In 2009, Takahashi et al., synthesised Trifarienols A (161) and B (162) from compound **165** (Scheme 25b) which was previously prepared by the group and attempted construction of the bicyclo [3.3.1]nonane ring system. HSR was employed to achieve product **165** as the sole product in 93% yield by treating intermediate **164** with ZnCl_2_ in chloroform. The entire synthetic process took 15 steps to accomplish 2% and 9% yields of Trifarienols A (**161**) and B (**162**), respectively (Scheme 25b) (Takahashi et al., 2009).

## 3 Conclusion

In summary, the HSR and its applications in the total synthesis of complex and contiguous bioactive organic molecules from natural sources, have been reviewed. The HSR is applied in the formation of C-C bonds and complex transformations via activated Lewis acid-promoted allylation of various electrophiles. HSR is a facile reaction that has enabled organic chemists to undertake key transformations for the formation of complex carbocyclic compounds. Examples of such transformation include hetero-Diels–Alder (HDA), allylation of aldehydes and ketones, allylation of cyclic oxonium cation, β,γ-unsaturated α-ketoesters, carbocyclization, allylation of imines, epoxide opening/allylsilylation, cyanation of carbonyls amongst others.

The versatility of the HSR has been enhanced following the various modifications to the original method to broaden the scope of its application and molecular transformations of both intermolecularly and intramolecularly. Modifications such as the use of homoallylic allowed the preparation of a wide range of compounds including acetals, aldimines, aldehydes, carboxylic acid chlorides, epoxides, ketals, ketoimines, ketones, and α,β-unsaturated carbonyl compounds. Furthermore, HSRs occur under mild conditions and have low turnover time, are typically high yielding as well as are diastereoselective, stereoselective and installing multiple stereogenic centres concurrently.

In this review, we describe the applications of HSR in the synthesis of key intermediates and final products from several natural and synthetic products.

## Glossary

AHSR: Asymmetric Hosomi-Sakurai reaction
AIBN: Azobisisobutyronitrile
AD: Alzheimer’s disease
aSH: aza-Hosomi-Sakurai
ATP: adenosine triphosphate
BINAP: 2,2′-bis(diphenylphosphino)-1,1′-binaphthyl
BPO: Benzoyl peroxide
CAB: Chiral (acyloxy)borane
cAMP: cyclic adenosine monophosphate
DCM: Dichloromethane
DIFLUOROPHOS: (2,2,2′,2′-Tetrafluoro-4,4′-bi-1,3-benzodioxole-5,5′-diyl)bis(diphenylphosphine)
DMF: Dimethylformamide
DMPU: N,N′-Dimethylpropyleneurea
DNA: Deoxyribonucleic Acid
**EC**_**50**_: half maximal effective concentration
**ED**_**50**_: median effective dose
GAP: GTPase-accelerating proteins
GETFund: Ghana Education Trust Fund
GNPC: Ghana National Petroleum Corporation
HDA: Hetero-Diels–Alder
HEK: human embryonic kidney cells
HFIP: Hexafluoroisopropanol
HIV: Human immunodeficiency virus
HSC: Hosomi-Sakurai cyclisation
HSR: Hosomi-Sakurai reaction
**IC**_**50**_: Half-maximal inhibitory concentration
LED: light-emitting diode
MOM: Methoxymethyl ether
NMR: Nuclear Magnetic Resonance spectroscopy
NOE: Nuclear Overhauser effect
RNA: Ribonucleic acid
STAT3: signal transducer and activator of transcription 3
TBAF: tetrabutylammonium fluoride
TBDPS: tert-butyldiphenylsilyl
TFE: trifluoroethanol
THF: tetrahydrofuran
TMS: trimethylsilyl
TMSI: trimethylsilyl iodide
TMSO: Trimethylsulfoxonium
TMSOT: Trimethylsilyl trifluoromethanesulfonate
