# Supplementary material for: Application of Hosomi-Sakurai allylation reaction in total synthesis of biologically active natural products
Source: Front Chem. 2025 Mar 28;13:1527387. doi: 10.3389/fchem.2025.1527387 (PMC11986726; doi:10.3389/fchem.2025.1527387)
Supplement: Supplementary file 1 [file Supplementaryfile1.docx]

**Electronic Supplementary Data**

**Application of Hosomi-Sakurai Allylation Reaction in Total Synthesis of Biologically Active Natural Products**

Justice Akwensi,^1^ Robert T. Kumah,^2^ Dorcas Osei-Safo,^1*^ Richard K. Amewu,^1*^

*^1*^Department of Chemistry, School of Physical and Mathematical Sciences, College of Basic and Applied Sciences, University of Ghana, Legon, Ghana.*

*^2^Department of Chemical and Petrochemical Engineering, School of Petroleum Studies, University of Mines and Technology, Tarkwa, Ghana.*

******Corresponding authors****:* [*ramewu@ug.edu.gh*](mailto:ramewu@ug.edu.gh)*,* [*dosei-safo@ug.edu.gh*](mailto:dosei-safo@ug.edu.gh)

**Table of contents**

| **Entry** | **Content** | **Page** |
| --- | --- | --- |
| 1 | **Scheme S1 - S12.** Reaction schemes showing HSR and its applications in total synthesis. | 1-3 |
| 2 | **Figures S1 and S2.** Structure of bioactive natural products and Ligands for preparing Lewis catalysts | 3-4 |

**Scheme S1.** Carbon-carbon bond-formation reactions (Lee, 2020).

**Scheme S2.** A mechanism of HSR of α-β-unsaturated carbonyl compounds (Hosomi *et al*., 1984).

**Scheme S3.** Hosomi-Sakurai allylation catalysed by non-recyclable catalyst.

**Scheme S4.** Allylation polymerization using the HSR (Itsuno, 2005).

**Scheme S5**. An asymmetric allylation reaction catalysed by (*R*)-DIFLUOROPHOS, (Komiyama *et al*., 2017; Wadamoto *et al*., 2003).

**Scheme S6.** The use of hexafluoroisopropanol (HFIP) as a source of hydrogen and a catalyst in the allylation of acetals (Berkessel *et al*., 2006).

**Scheme S7.** Application of trityl tetrakis (pentafluorophenyl) borate as Lewis acid in HSR β,γ-unsaturated α-ketoesters to yield γ,γ-disubstituted α-ketoesters (Gan *et al*., 2022).

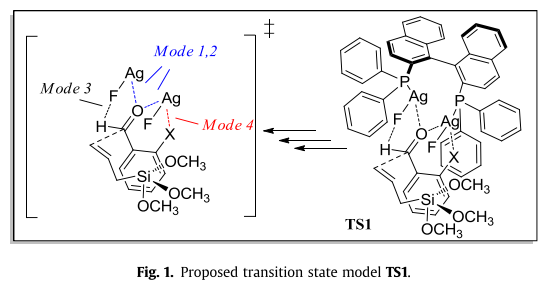


**Scheme S8**. Proposed transition state for Sakurai-Hosomi-Yamamoto allylation reaction (Mirabdolbaghi & Dudding, 2012; Yamamoto & Asao, 1993).

**Scheme S9**. Total synthesis of Deoxopinguisone (Tori *et al.,* 2001).

**Scheme S11.** Intramolecular cyclisation *via* the HSR transition state (Uyehara *et al.,* 1985, 1986).

**Scheme S12**. Total synthesis of Paniculatine, **S35** involving HSR step (Liu *et al*., 2019).

**Scheme S13**. Enantioselective coupling of aldehydes using photocatalyst (Schäfers et al., 2022).

**Figure S1**. Examples of chiral Lewis acids and base catalyst (Schäfers *et al*., 2022).

**Figure S2.** Structure of enyne-containing natural products.

**References**

Berkessel, A., Adrio, J. A., Hüttenhain, D., & Neudörfl, J. M. (2006). Unveiling the “booster effect” of fluorinated alcohol solvents: Aggregation-induced conformational changes and cooperatively enhanced H-bonding. *Journal of the American Chemical Society*, *128*(26), 8421–8426. https://doi.org/10.1021/ja0545463

Gan, Z., Cui, D., Zhang, H., Feng, Y., Huang, L., Gui, Y., Gao, L., & Song, Z. (2022). Trityl Cation-Catalyzed Hosomi-Sakurai Reaction of Allylsilane with β,γ-Unsaturated α-Ketoester to Form γ,γ-Disubstituted α-Ketoesters. *Molecules*, *27*(15), 4730–4741. https://doi.org/10.3390/molecules27154730

Hosomi, A., Sakata, Y., & Sakurai, H. (1984). N-(trimethylsilylmethyl)aminomethyl ethers as azomethine ylide synthons. A new and convenient access to pyrrolidine derivatives. *Chemistry Letters*, *13*(7), 1117–1120. <https://doi.org/10.1246/cl.1984.1117>

Itsuno, S. (2005). Chiral polymer synthesis by means of repeated asymmetric reaction. *Progress in Polymer Science (Oxford)*, *30*(5), 540–558. https://doi.org/10.1016/j.progpolymsci.2005.01.008

Komiyama, T., Minami, Y., & Hiyama, T. (2017). Recent Advances in Transition-Metal-Catalyzed Synthetic Transformations of Organosilicon Reagents. *ACS Catalysis*, *7*(1), 631–651. <https://doi.org/10.1021/acscatal.6b02374>

Lee, J. H. (2020). Use of the Hosomi-Sakurai allylation in natural product total synthesis. *Tetrahedron*, *76*(33), 131351–131367. https://doi.org/10.1016/j.tet.2020.131351

Liu, J., Chen, S., Li, N., & Qiu, F. G. (2019). A Concise Total Synthesis of (+)-Paniculatine. *Advanced Synthesis and Catalysis*, *361*(15), 3514–3517. https://doi.org/10.1002/adsc.201900376

Mirabdolbaghi, R., & Dudding, T. (2012). A catalytic asymmetric approach to C 1-chiral 3-methylene-indan-1-ols. *Tetrahedron*, *68*(7), 1988–1991. https://doi.org/10.1016/j.tet.2011.12.040

Schäfers, F., Dutta, S., Kleinmans, R., Mück-Lichtenfeld, C., & Glorius, F. (2022). Asymmetric Addition of Allylsilanes to Aldehydes: A Cr/Photoredox Dual Catalytic Approach Complementing the Hosomi-Sakurai Reaction. *ACS Catalysis*, *12*(19), 12281–12290. <https://doi.org/10.1021/acscatal.2c03960>

Tori, M., Makino, C., Hisazumi, K., Sono, M., & Nakashima, K. (2001). Synthesis of a homochiral ketone having a pinguisane skeleton using phenylethylamine as a chiral auxiliary: A formal total synthesis of deoxopinguisone. *Tetrahedron Asymmetry*, *12*(2), 301–307. https://doi.org/10.1016/S0957-4166(01)00041-6

Uyehara, T., Kabasawa, Y., Kato, T., & Furuta, T. (1985). Photochemical rearrangement approach to the total synthesis of (±)-pinguisone and (±)-deoxopinguisone. *Tetrahedron Letters*, *26*(19), 2343–2346. https://doi.org/10.1016/S0040-4039(00)95094-9

Wadamoto, M., Ozasa, N., Yanagisawa, A., & Yamamoto, H. (2003). BINAP/AgOTf/KF/18-crown-6 as new bifunctional catalysts for asymmetric Sakurai-Hosomi allylation and Mukaiyama aldol reaction. *Journal of Organic Chemistry*, *68*(14), 5593–5601. https://doi.org/10.1021/jo020691c
